# Assessing the impact of Benzo[*a*]pyrene on Marine Mussels: Application of a novel targeted low density microarray complementing classical biomarker responses

**DOI:** 10.1371/journal.pone.0178460

**Published:** 2017-06-26

**Authors:** Mohamed Banni, Susanna Sforzini, Volker M. Arlt, Audrey Barranger, Lorna J. Dallas, Caterina Oliveri, Yann Aminot, Beniamina Pacchioni, Caterina Millino, Gerolamo Lanfranchi, James W. Readman, Michael N. Moore, Aldo Viarengo, Awadhesh N. Jha

**Affiliations:** 1 Department of Sciences and Technological Innovation (DiSIT), University of Piemonte Orientale "A. Avogadro", Alessandria, Italy; 2 Laboratory of Biochemistry and Environmental Toxicology, ISA chott-Mariem, Sousse University, Sousse, Tunisia; 3 Analytical and Environmental Sciences Division, King's College London, MRC-PHE Centre for Environmental & Health, London, United Kingdom; 4 NIHR Health Protection Research Unit in Health Impact of Environmental Hazards at King’s College London in partnership with Public Health England, London, United Kingdom; 5 School of Biological and Marine Sciences, University of Plymouth, Plymouth, United Kingdom; 6 Centre for Chemical Sciences, University of Plymouth, Plymouth, United Kingdom; 7 University of Padova, Padova, Italy; 8 Plymouth Marine Laboratory, Prospect Place, The Hoe, Plymouth, United Kingdom; 9 European Centre for Environment & Human Health (ECEHH), University of Exeter Medical School, Knowledge Spa, Royal Cornwall Hospital, Truro, Cornwall, United Kingdom; University of Siena, ITALY

## Abstract

Despite the increasing use of mussels in environmental monitoring and ecotoxicological studies, their genomes and gene functions have not been thoroughly explored. Several cDNA microarrays were recently proposed for *Mytilus* spp., but putatively identified partial transcripts have rendered the generation of robust transcriptional responses difficult in terms of pathway identification. We developed a new low density oligonucleotide microarray with 465 probes covering the same number of genes. Target genes were selected to cover most of the well-known biological processes in the stress response documented over the last decade in bivalve species at the cellular and tissue levels. Our new ‘STressREsponse Microarray’ (STREM) platform consists of eight sub-arrays with three replicates for each target in each sub-array. To assess the potential use of the new array, we tested the effect of the ubiquitous environmental pollutant benzo[*a*]pyrene (B[a]P) at 5, 50, and 100 μg/L on two target tissues, the gills and digestive gland, of *Mytilus galloprovincialis* exposed *invivo* for three days. Bioaccumulation of B[a]P was also determined demonstrating exposure in both tissues. In addition to the well-known effects of B[a]P on DNA metabolism and oxidative stress, the new array data provided clues about the implication of other biological processes, such as cytoskeleton, immune response, adhesion to substrate, and mitochondrial activities. Transcriptional data were confirmed using qRT-PCR. We further investigated cellular functions and possible alterations related to biological processes highlighted by the microarray data using oxidative stress biomarkers (Lipofuscin content) and the assessment of genotoxicity. DNA damage, as measured by the alkaline comet assay, increased as a function of dose.DNA adducts measurements using ^32^P-postlabeling method also showed the presence of bulky DNA adducts (i.e. dG-*N*^2^-BPDE). Lipofiscin content increased significantly in B[*a*]P exposed mussels. Immunohistochemical analysis of tubulin and actin showed changes in cytoskeleton organisation. Our results adopting an integrated approach confirmed that the combination of newly developed transcriptomic approcah, classical biomarkers along with chemical analysis of water and tissue samples should be considered for environmental bioimonitoring and ecotoxicological studies to obtain holistic information to assess the impact of contaminants on the biota.

## Introduction

In the last two decades, genomics-based approaches have provided insights into the mechanisms underlying the biological responses in natural biota following exposures to environmental pollutants. Recent developments in ‘omics’ technologies have raised our expectations to elucidate crucial biological processes following exposure to pollutants to which biota are particularly exposed chronically at low dose/ concentrations. Notably, transcriptomics, the simultaneous measurement of thousands of mRNAs in a biological sample, has been a dependable tool, increasing our understanding of many important physiological processes in marine biosystems [1,2,3,4,5]. The accessibility of new genomic resources, high-throughput molecular technologies such as microarray, pyrosequencing and next generation sequencing have provided information and helped to identify molecular targets of pollutants (e.g. genes) that contribute to stress in natural populations [6,7].

Marine mussels belonging to the genus *Mytilus* are commonly found in the coastal areas of the Mediterranean and Atlantic Ocean and play a significant role in coastal ecology apart from being economically important for shellfish industries [8]. *Mytilus* spp. have been used extensively in biomonitoring projects through the application of a battery of physiological and cellular markers, providing evidence of a stress syndrome and demonstrating the biological risk associated with polluted environments [9,10]. Mussels are particularly useful in this context because they inhabit regions of different pollution status, accumulate xenobiotics, and are sessile. However, despite the increasing use of these organisms in an ecotoxicological context, their genomes and gene functions have not been thoroughly investigated. Although several cDNA microarrays were developed recently in *Mytilus galloprovincialis*, *Mytilus edulis*, and *Mytilus trossulus*, putatively identified partial transcripts limit their use in generating reliable gene expression profiles in terms of pathway identification[11].

Benzo[*a*]pyrene (B[a]P) is a known genotoxic and carcinogenic agent and a ubiquitous environmental contaminant. It is one of the US EPA’s priority pollutants. As one of the most commonly tested compounds in ecotoxicology [12,13], B[a]P has been shown to produce toxic effects in aquatic organisms across a range of biological levels. For example, B[a]P induces xenobiotic metabolism in fish and marine polychaetes [14,15], causes developmental abnormalities and genotoxic effects in the embryos of oysters, annelids and zebrafish[16,17,18]. B[a]P also has the ability to form genotoxic intermediates, such as B[a]P-7,8-dihydrodiol-9,10-epoxide (BPDE), which form bulky DNA adducts that may result in mutation [19,20], with potential impacts on the individual organism or population [21, 22]. B[a]P has also been reported to induce genotoxicity, oxidative stress [12,23,24] and endocrine disruption [25] in marine bivalves. In addition, B[a]P has been shown to interact with other pollutants (e.g. engineered nanoparticles) that could alter the potential toxic effects [13,26,27]. As such, mixture effects become more prominent in ecotoxicology and environmental protection [28].A better understanding of the mechanisms underlying the toxicity of this ubiquitous contaminant would allow us to assess its role in more realistic, multi-component toxicity.

In the backdrop of the above information, our study adopts an integrated approach, presenting for the first time, a new low density oligonucleotide microarray in mussel species complementing other novel biomarker responses in an ecotoxicological context. Target genes used in the new platform were selected to cover most of the well-known biological processes including covering stress related responses in bivalve species(e.g. lysosomal activity, DNA metabolism, translation, mitochondrial activity, carbohydrate metabolism, heat shock response, oxidative stress, proteolysis, phase I and II xenobiotic metabolism, metal resistance, endocrine disruption, immune response, pumps, cytoskeleton, and adhesion to substrate).The new platform was used to evaluate the biological effects of exposure to three concentrations of B[a]P on the gills and digestive gland (DG) of *M*. *galloprovincialis*. Q-RT-PCR analysis was performed to confirm the over-represented biological processes in response to B[a]P exposure (i.e., DNA metabolism, oxidative stress, and cytoskeleton). An important aspect of this study was to investigate the consequences of transcriptomic changes depicted by microarray data on relevant biological processes in gills and DG at the cellular/tissue level. To complement the microarray and transcriptomics responses, genotoxic responses were assessed by determining DNA strand breaks using the comet assay and determination of bulky DNA adduct formation by^32^P-postlabelling method. The presence of oxidative stress was evaluated by lysosomal lipofuscin accumulation and the alteration of cytoskeletal structures by immuno-histochemistry. Concentration of B[a]P in water and tissue samples were also determined using analytical chemistry techniques.

## Materials and methods

### Microarray development

The STressREsponse Microarray (STREM) was designed using available annotated sequences from *Mytiluscalifornianus*[11], *Mytilus galloprovincialis*[29], *M*. *edulis*[1], and *Crassostera gigas*[1]. 465 Target genes were selected to cover 15well-known biological processes in the stress response documented over the last decade in bivalve species [29,12,3,30,4,2].

### Chemicals and organisms

Unless otherwise stated, all chemicals were purchased from Sigma-Aldrich (UK/Italy). Adult *M*. *galloprovincialis* (50.7 ± 2.8 mm) were collected from the intertidal zone at Trebarwith Strand, Cornwall, UK (50° 38’ 40" N, 4° 45’ 44" W) in October 2014. No specific permission is required to collect marine mussels which are abundant at this collection site in Cornwall, UK. Collection of the limited number of mussels for laboratory based experiments does not have any detrimental effects on the environment at the site and does not involve any ethical issues. It does not require any licence from any authorities in the UK as they are not protected or endangered species. This is in line with our previous studies using this species collected from this site [12,13].This site has been used previously as a reference location for ecotoxicological studies [31]. The site is relatively free of disease and is remotely located [32]. Mussels were transported back to the laboratory in cool boxes and allowed to depurate for a minimum of 1 week in natural seawater from Plymouth Sound. During this period, mussels were fed a suspension of *Isochrysis galbana* every 3 days (1.05 × 10^6^ cells/mL), with a 100% water change 2 hours after each feeding [33].

### Experimental design and sampling

After depuration, the mussels were transferred to 2-L glass beakers containing 1.8 L of the same seawater as above and allowed to acclimatize for 48 h. The experiment began after this period and consisted of a 3-day static exposure with no water changes, during which the mussels were not fed. Two mussels were used per beaker. A photoperiod of 12h light: 12h dark was maintained throughout the experiment. Good seawater oxygenation was provided by a bubbling system. Seawater quality was monitored in each of the beakers by measuring salinity (35.4 ± 0.09‰), pH (7.9 ± 0.06), %dissolved oxygen (97.9 ± 3.22%) and temperature (15.3 ± 0.68°C).Groups of mussels were exposed to five treatments: a solvent control (0.02% dimethyl sulfoxide [DMSO]; 36 mussels); 5 μg/L B[a]P (36 mussels); 50 μg/L B[a]P (36 mussels); 100 μg/L B[a]P (36 mussels); and 1000 μg/L B[a]P (9 mussels). After 3 days, mussel sex was determined by mantle smear and light microscopy. Females were processed for transcriptomics (microarray and qRT-PCR) and immunohistochemistry (DG only). Individuals of both sexes were used for the assessment of genotoxicity (i.e. DNA damage measured by the comet assay or bulky DNA adducts by ^32^P-postlabelling; gills and DG). Tissue samples were also taken from the DG and gills for chemical analysis of the B[a]P concentrations using gas chromatography-mass spectrometry (GC-MS). Samples were transferred into pre-weighed glass vials and frozen for storage awaiting analysis.

### Determination of B[a]P concentrations in water and tissues by GC-MS

Water samples (9 mL) were collected in glass vials and dichloromethane (1 mL, HPLC grade, Rathburn Chemicals Ltd., UK) was added before storage in the dark at 4°C [13]. Prior to analysis, B[a]P d12 (15 μL of a 3 mg/L solution in acetone) was spiked as an internal standard. Following thorough shaking, the dichloromethane layers were removed into glass injection vials.

Before analysing the tissue samples(Gills and DG), water was removed by freeze drying (Christ Alpha LDplus freeze-dryer), typically for 24 h, and the water content determined by differential weighing. Dried tissue was ground in the vials using a razor blade and was spiked with 50 μL of the internal standard. Dichloromethane (2 mL) was added and the tissues extracted in a sonication bath for 20 min. The extracts were filtered through pre-cleaned glass wool. The lipid content was determined by sub-sampling a weighed aliquot of 500 μL and drying it in a nitrogen evaporator. The dry weight was considered to be the lipid content[34].

Both water and tissue extracts were analysed using an Agilent Technologies 7890A Gas Chromatography (GC) system interfaced with an Agilent 5975 series Mass Selective(MS) detector. A Restek Rxi-1MS (crosslinked dimethyl polysiloxane) capillary column (30 m) with a film thickness of 0.25 μm and internal diameter of 0.25 mm was used for separation, with helium as a carrier gas (maintained at a constant flow rate of 1 mL/min). Extracts were injected splitless, with the injector maintained at 300°C. The oven temperature was 40°C for 1 minute and then increased at 15°C/minute to a final temperature of 300°C, which was held for 4 minutes. The mass spectrometer was operated in electron impact mode at 70 eV with the ion source and quadrupole analyzer temperatures fixed at 230°C and 150°C, respectively. Samples were screened for B[a]P and B[a]P d12 using selected ion monitoring in which the target ions were 252 (253 and 126 for confirmation purposes) and 264, respectively. Within each batch of samples, multiple solvent blanks and standard calibrating mixtures were run regularly to ensure the stability of the system performance. Procedural blanks were also included in the sequence for quality assurance purposes. The B[a]P concentration was calculated based on the internal standard. Often, due to their high lipid content, mussel extracts can require a preliminary sample clean-up prior to GC-MS. In our case, the B[a]P signal intensities for the exposed mussels were sufficiently high to avoid this additional step.

### Microarray hybridization and analysis

Competitive dual-color microarray hybridization was performed with the new STREM platform; fluorescence-labeled cDNA probes were obtained by direct labeling in the presence of modified Cy3- and Cy5-dCTP (Perkin Elmer). The procedure was carried out as described previously [3] using 0.5 μg of an anchored oligodT(19)VN. Total RNA was extracted from pieces of individual tissues(gills and DG) from females using acid phenol-chloroform precipitation according to [35] with TRI-Reagent (Sigma-Aldrich).Four biological replications were considered for the microarray and for the qRT-PCR analysis. The RNA was further purified by precipitation in the presence of 1.5 M LiCl_2_, and the quality of each RNA preparation was confirmed by UV spectroscopy and TBE agarose gel electrophoresis in the presence of formamide [3]. Laser scanning of microarrays was performed with an Agilent G2565CA scanner (Agilent Technologies, Inc., USA) at 5-μm resolution. Sixteen-bit TIFF images were analyzed using Genepix 6.0 (Axon) to extract raw fluorescence data from each spot.

We performed global mean normalization across microarray surfaces and local mean normalization across element signal intensity. Log2 transformation was performed for each expression level. Each experimental condition was evaluated as 12 separate values (3 technical and 4 biological replicates). Differentially expressed genes were identified by Significance Analysis of Microarray (SAM, http://statweb.stanford.edu/~tibs/SAM/). Gene expression was considered to be significantly different in the test condition versus the reference condition when the log value was > 0.7.

The experimental design accounted for a complete “triangular loop” in which each RNA sample from the tissue taken from mussels exposed to the three B[a]P conditions was hybridized with RNA from control mussels (DMSO-exposed mussels). Each experimental condition had at least four biological replicates from individual female animals using the day-swap procedure, for a total of 24 experiments. MIAMI-compliant microarray data, including a detailed description of the experimental design and each hybridization experiment, were deposited in the Gene Expression Omnibus (http://www.ncbi.nlm.nih.gov/geo/query/) under identifier GSE84605.

### Functional genomics analysis

Functional characterization of mussel genes represented on a microarray was based on Gene Ontology (GO) annotation and carried out in Blast2GO [36] using default parameters. However, in the case of the STREM platform, target genes were putatively annotated and ranked under established biological processes, making the generation of robust processes easier and faster.

### qRT-PCR

The qRT-PCR was carried out using the same RNA extract as microarray hybridization. Relative mRNA abundance of the mussel genes encoding 10 Probes and primer pairs (S1 Table) were designed using Beacon Designer v3.0 (Premier Biosoft International, Inc.). All primers and dual-labeled TaqMan probes were synthesized by MWG-Biotech GmbH (Germany).

The cDNA (25 ng RNA reverse-transcribed to cDNA) was amplified in a CFX384 Real-Time PCR detection system (Bio-Rad Laboratories) with iQTM Multiplex Power mix (Bio-Rad Laboratories) according to the manufacturer’s instructions for the triplex protocol. All multiplex combinations accounted for the following dual fluorescence tags: 6-carboxyfluorescein/Black Hole (BH) 1; 6-carboxy-2′,4,4′,5′,7,7′-hexachlorofluorescein/BH1; and Texas Red/BH2. Briefly, cDNA was amplified in a final reaction volume of 10 μL including 1X iQTM Multiplex Power mix, 0.3 μmol/L of each primer, and 0.1 μmol/L of each probe (S1 Table). Relative expression data were geometrically normalized to 18S rRNA (L33452), an invariant actin isotype (AJ625116), and ribosomal protein riboL27 (AJ625928), which were selected from a list of genes for which expression does not vary over more than 50 conditions, including toxic treatments, stages of the life cycle, and various tissues [3].

A specific duplex TaqMan assay was developed to amplify 0.25 ng of RNA reverse-transcribed to cDNA in the presence of 0.1 μmol/L of each dual-labeled probe (hexachlorofluorescein/BH1 for actin and Texas Red/BH2 for 18S rRNA) and 0.1 μmol/L and 0.4 μmol/L of forward and reverse primer, respectively, for 18S rRNA and actin (S1 Table). For all TaqMan assays, the reaction occurred as follows: 30 seconds at 95°C, followed by 40 cycles of 10 seconds at 95°C and 20 seconds at 60°C. The qRT-PCR was performed with four biological replicates and three technical replicates. For the assays P53,Topoisomerase and DNA ligase, the thermal protocol was as presented by [3]. Statistical analyses were carried out on the group mean values using a random reallocation test[37].

### Determination of DNA strand breaks using the comet assay

Gill and DG tissues were stored on ice prior to processing for the comet assay. After rough chopping, each piece of tissue was added to 1 mL of 1.6 mg/mL dispase II solution (in Hank’s buffered saline) pre-warmed to 37°C. After digesting in the dark for 30 min, the resulting suspension was coarsely filtered through gauze and spun at 200 *g* to remove any debris. The supernatant was checked for cell viability using trypan blue (0.04%), and only samples with >90% unstained cells were used. A sub-sample of 100 μL of the cell suspension was pelleted at 350 g and 180 μL low melting point agarose added. Two replicate microgels (75 μL) were formed by coverslipping and allowed to set at 4°C for a minimum of 15 min. After the cover slips were removed, cells were lysed in 2.5 M NaCl, 100 mM EDTA, 10 mMTris, 1% N-lauryl-sarcosine, 1% Triton X-100, and 10% DMSO (pH adjusted to 10 with NaOH) for 1 h at 4°C in the dark. Slides were then transferred to an electrophoresis chamber containing 1 mM EDTA and 0.3 M NaOH (pH 13). DNA was allowed to unwind in the dark for 20 min, followed by electrophoresis at 1 V/cm (∼300 mA) for 20 min in the dark. Gels were preserved with ice cold 100% ethanol and then scored after the addition of 20 μg/mL ethidium bromide using a Leica epifluorescence microscope (Leica Microsystems, Milton Keynes, UK) and Comet 4 image analysis system (Perceptive Instruments, Bury St Edmunds, UK). One hundred nucleoids were assessed per slide; all samples were measured blind. Tail intensity (% tail DNA), defined as the percentage of DNA migrated from the head of the comet into the tail, was used as a measure of DNA damage induced.

### Determination of DNAadduct formation using ^32^P-postlabelling method

DNA was isolated from gills and DG tissue using a standard phenol-chloroform extraction procedure as used previously in mussels [38]. We used the nuclease P1 enrichment version of the thin-layer chromatography (TLC) ^32^P-postlabelling assay [39] to detect B[a]P-derived DNA adducts (i.e. 10-(deoxyguanosin-*N*^2^-yl)7,8,9-trihydroxy-7,8,9,10-tetrahydro-B[a]P [dG-*N*^2^-BPDE]). The procedure was essentially as described previously [39]; DNA samples (4 μg) were digested with micrococcal nuclease (288 mU; Sigma) and calf spleen phosphodiesterase (1.2 mU; MP Biomedical), and then enriched and labelled as reported [40]. Solvent conditions for the separation of B[a]P-derived DNA adducts were as follows: D1, 1.0 M sodium phosphate, pH 6.0; D3, 3.5 M lithium-formate, 8.5 M urea, pH 3.5; D4, 0.8 M lithium chloride, 0.5 M Tris, 8.5 M urea, pH 8.0. After chromatography, TLC sheets were scanned using a Packard Instant Imager (Dowers Grove, IL, USA) and DNA adduct levels (RAL, relative adduct labelling) were calculated as reported [41]. An external BPDE-modified DNA standard was used as a positive control [19].

### Lysosomal lipofuscin content

Lysosomal lipofuscin content in the cells of the digestive glands was estimated on cryostat sections (10 μm) obtained as previously reported [42]. The lipofuscin content was assessed using the Schmorl reaction [43]. Lipofuscin accumulation was quantified by image analysis as described above and expressed as a percentage variation with respect to controls. Data were analyzed by the non-parametric Mann-Whitney *U*-test.

#### Immunofluorescence analysis

For histochemistry, tissues were mounted on aluminum chucks, frozen in super-cooled n-hexane and stored at—80 ° C. Frozen digestive gland sections (10 μm) of ten mussels from each exposure condition were cut by cryostat (Leica CM3050) and flash-dried by transferring them onto poly-L-lysine-coated microscope slides at room temperature. Digestive gland tissue sections were fixed in PFA solution (4% in PBS, pH 7.2) for 20 min at 20 ± 1 °C.

Immunofluorescent anti-tubulin staining: after fixation, sections were washed three times in PBS (5 min) and incubated in a permeabilisation and blocking solution (0.5% Triton X-100, 2% bovine serum albumin-BSA, 0.5% goat serum in PBS) for 1 h at 20 ± 1°C. After rinsing, sections were incubated with the primary antibody (rabbit polyclonal to tubulin, Abcam, 1/100 in PBS containing 1% BSA and 0.05% Triton X-100) overnight at 4°C, and then in the secondary antibody i.e. goat anti-rabbit IgGH&L (Chromeo™ 488) (Abcam) (1/100 in 1% BSA and 0.05% Triton X-100 in PBS) for 1 h at 20 ± 1°C in the dark. Sections were then rinsed in PBS and then mounted in Mowiol mounting medium (Cold Spring HarbProtoc, 2006). Controls for non-specific staining included sections that were processed in the absence of the secondary antibodies. Slides were viewed under 400 x magnification by an inverted photo-microscope (Zeiss Axiovert 100M connected to a digital camera Zeiss AxioCamMRm) equipped for fluorescence microscopy using FITC emission filter [44]. Images were analysed using an image analysis system (Scion Image) that allowed for the quantification of the mean fluorescence intensity. Data were analysed by the non-parametric Mann-Whitney *U*-test.

F-actin staining: after fixation, sections were washed three times in PBS (5 min) and incubated in a permeabilisation solution (0.5% Triton X-100) for 1 h at 20 ± 1 °C. After rinsing, sections were incubated with Green Fluorescent Phalloidin Conjugate (CytoPainter F-actin Labeling Kit-Green Fluorescence—Abcam) for 1 h at 20 ± 1 °C. Sections were then rinsed in PBS and then mounted in Mowiol mounting medium. Slides were viewed under 400 x magnification by an inverted photo-microscope (Zeiss Axiovert 100M connected to a digital camera Zeiss AxioCamMRm) equipped for fluorescence microscopy using a FITC emission filter.

## Results

### B[a]P in water and tissue samples

The performance of the chemical analyses was determined by calculating the recovery after extracting spiked samples. Water samples were spiked with B[a]P at 4.3 and 33 μg/L in triplicate and extracted following the protocol described in the Methods section. The method was shown to be accurate and repeatable with recoveries of 99 ± 2% and 102 ± 4%, respectively. Regarding tissue analyses, gills and DG from unexposed mussels were spiked with B[a]P at 10 and 50 μg/g dry weight, respectively. Recoveries of 99 ± 3% and 104 ± 3% for the gills and digestive gland (DG), respectively, indicate accuracy and repeatability, and that the mussel’s high lipid content had no matrix effects on the analyses at these selected concentrations.

Measured water concentrations of B[a]P at time 0were in good agreement with nominal values at 63%, 104% and 121% for 5, 50, and 100 μg/L, respectively, The B[a]P concentration in water did not change significantly between 0 and 1 h, but decreased to approximately 10% of their initial values within 24 h (S1 Fig).

The B[a]P concentration in both DG and gill tissues was below the limit of detection for control mussels (<0.5 μg g^-1^ dry weight)and increased with the concentration of exposure (Table 1). In addition, the lipid concentration also increased with increasing exposure concentrations for both tissues. In gills, the water content was higher and the lipid content was lower than in DG.

**Table 1:** Chemical analysis of mussel tissues, after exposure to 0–100 μg L-1 BaP for 3 days.

	BaP concentration (μg L^-1^)			
Treatment	0	5	50	100
***Digestive gland***				
Dry weight (mg)	52	68	70	55
Water content (% wet weight)	76	76	76	75
Lipid content (mg g^-1^ dry weight)	52	74	154	135
BaP concentration (μg g^-1^ dry weight)	< 0.5	6.4	58.3	137.7
***Gills***			
Dry weight (mg)	35	41	29	23
Water content (% wet weight)	92	91	91	95
Lipid content (mg g^-1^ dry weight)	30	45	58	82
BaP concentration (μg g^-1^ dry weight)	< 0.5	16.0	100.4	253.6

### Microarray architecture

A final set of 15 biological processes at the cellular and tissue levels were retained: lysosomal activity, DNA metabolism, translation, mitochondrial activity, carbohydrate metabolism, heat shock response, oxidative stress, proteolysis, phase I and II xenobiotic metabolism, metal resistance, endocrine disruption, immune response, pumps, cytoskeleton, and adhesion to substrate. A minimum of 10 and a maximum of 80 target genes were considered for each biological process depending on the available annotated sequences (S2 Table). All probes were designed starting from complete or partial cDNA sequences. Oligo probes (60-mer) were designed for each target gene, covering the 3’ region of the mRNA sequence and with particular attention on guaranteeing optimal gene specificity in the case of homologous genes, such as metallothionein isotypes. A total of 465 probes (S2 Table) that were triplicated randomly to fill a 1395 feature sub-microarray for a total of eight sub-arrays per slide (S2 Fig) spotted by MicroCRIBI Service (Università di Padova, Italy).

### Microarray test and applications

**Fig 1:**
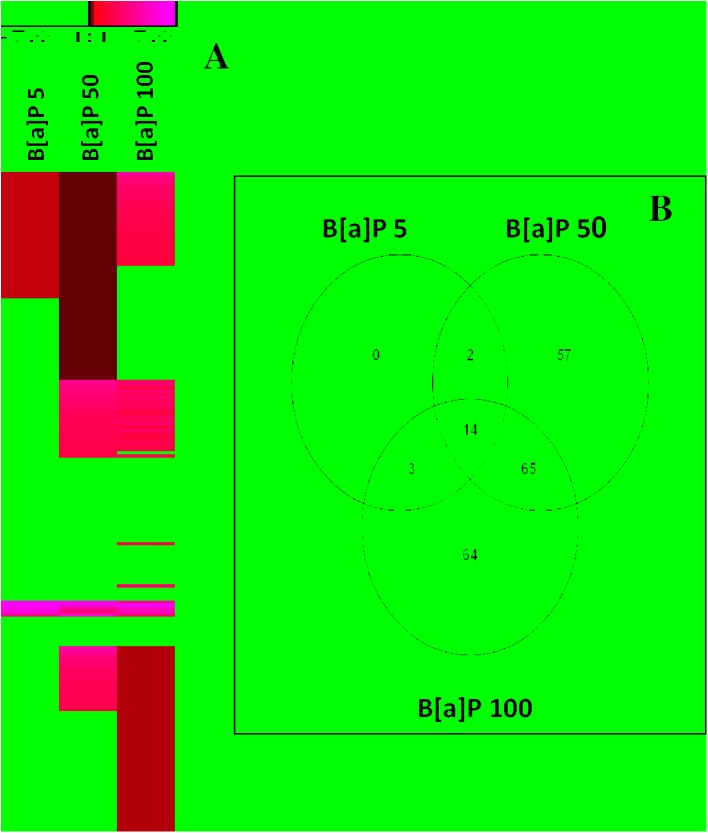
*Mytilus galloprovincialis* gene expression profiles of gills tissue in animals exposed to B[a]P (5μg/L, 50μg/L and 100 μg/L). The heat map (A) (Pearson correlation, complete linkage algorithm) reports the log2 relative expression level with respect to the reference condition. 205 differentially expressed genes were generated in at least one condition. Microarray data were analyzed using the Linear Mode for Microarray Analysis (LIMMA) software. B statistics with adjusted p value, 0.05 and B.0 were used as threshold for rejection of the null hypothesis (no variation). Supporting information to Fig 2 is presented in S2 Table and S3 Table. Venn diagram representation of gene expression patterns (Panel B) clearly depicted that only 14 DEGs are shared between the three experimental conditions. All DEGs are obtained respect to the control condition. Data used to generate the Venn-diagram were obtained from microarray analysis (S3 Table). Four biological replications of were considered.

**Fig 2:**
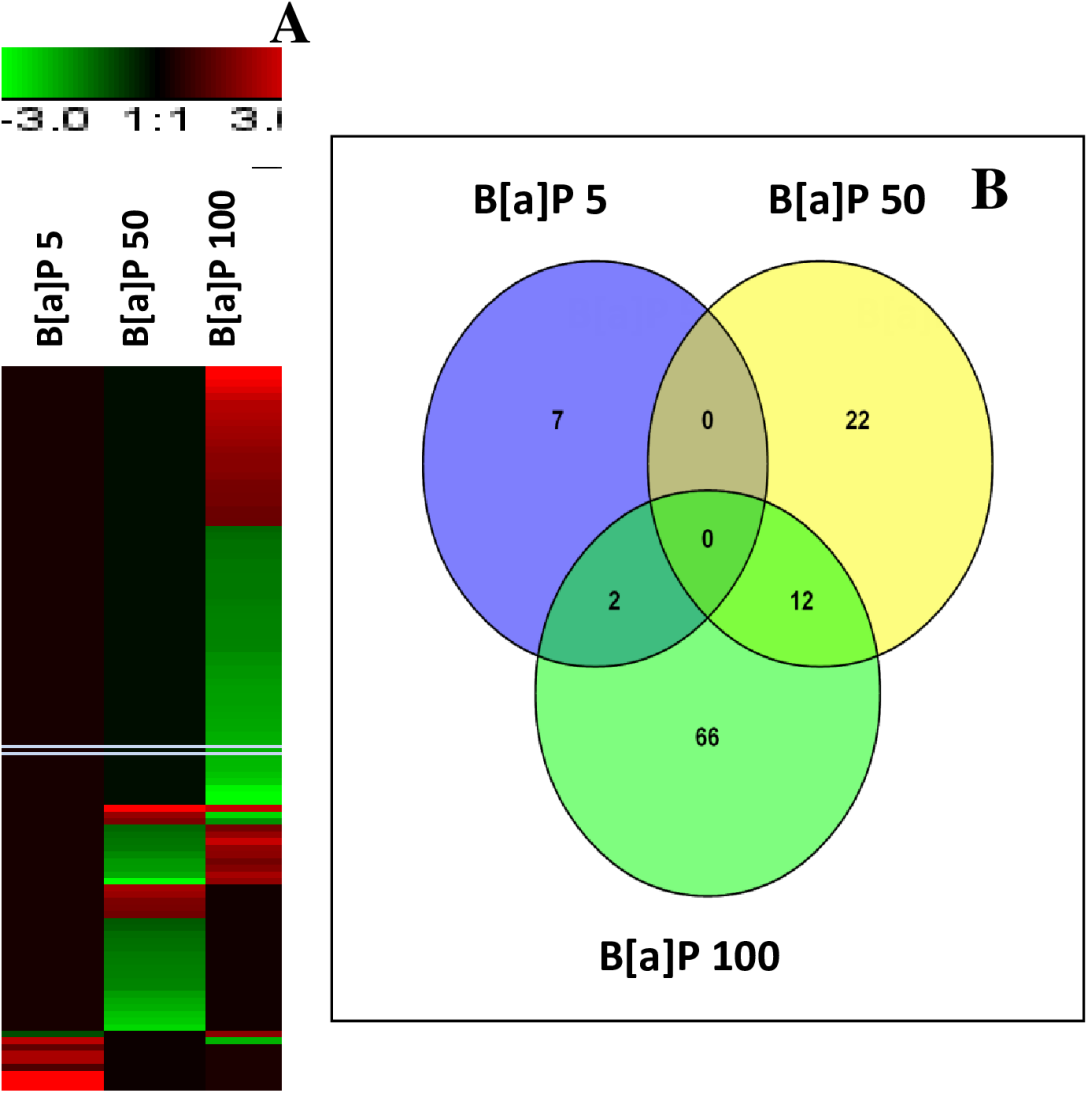
*Mytilus galloprovincialis* gene expression profiles of digestive gland tissue in animals exposed to B[a]P (5μg/L, 50μg/L and 100 μg/L). The heat map (A) (Pearson correlation, complete linkage algorithm) reports the log2 relative expression level with respect to the reference condition. 109 differentially expressed genes were generated in at least one condition. Microarray data were analyzed using the Linear Mode for Microarray Analysis (LIMMA) software. B statistics with adjusted p value, 0.05 and B.0 were used as threshold for rejection of the null hypothesis (no variation). Supporting information to Fig 3 is presented in S2 Table and S4 Table. Venn diagram representation of gene expression patterns (Panel B) clearly depicted that no DEGs are shared between the three experimental conditions. All DEGs are obtained respect to the control condition. Data used to generate the Venn-diagram were obtained from microarray analysis (S4 Table). Four biological replications of were considered.

**Fig 3:**
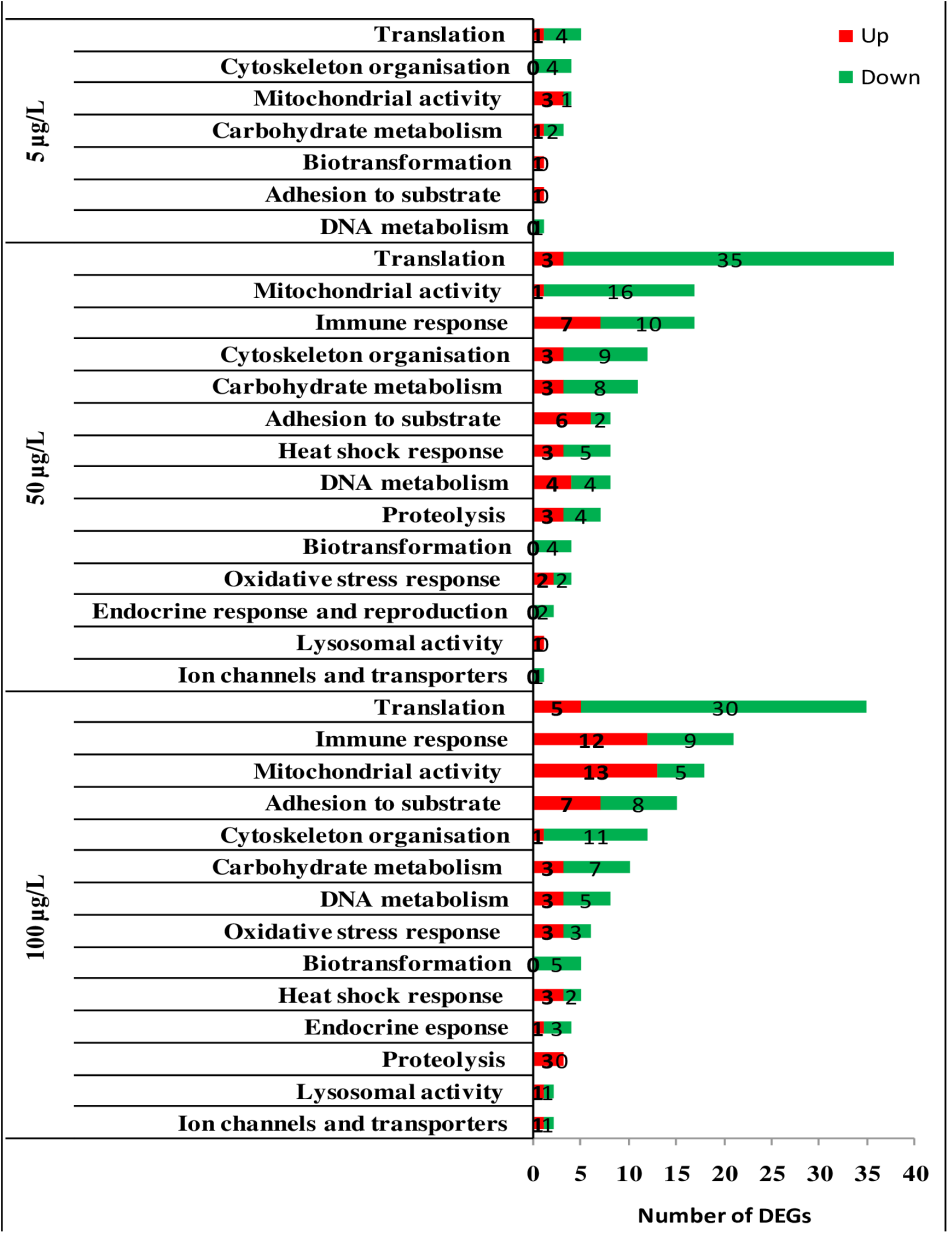
Over-representation analysis of DEGs in the gills tissue of mussels exposed to B[a]P (5μg/L, 50μg/L and 100 μg/L). Showed are: experimental condition; biological processes; Number of up-regulated genes; Number of down-regulated genes. The over-represented biological processes in B[a]P-exposed animals *versus* control. (S3 Table).

**Fig 4:**
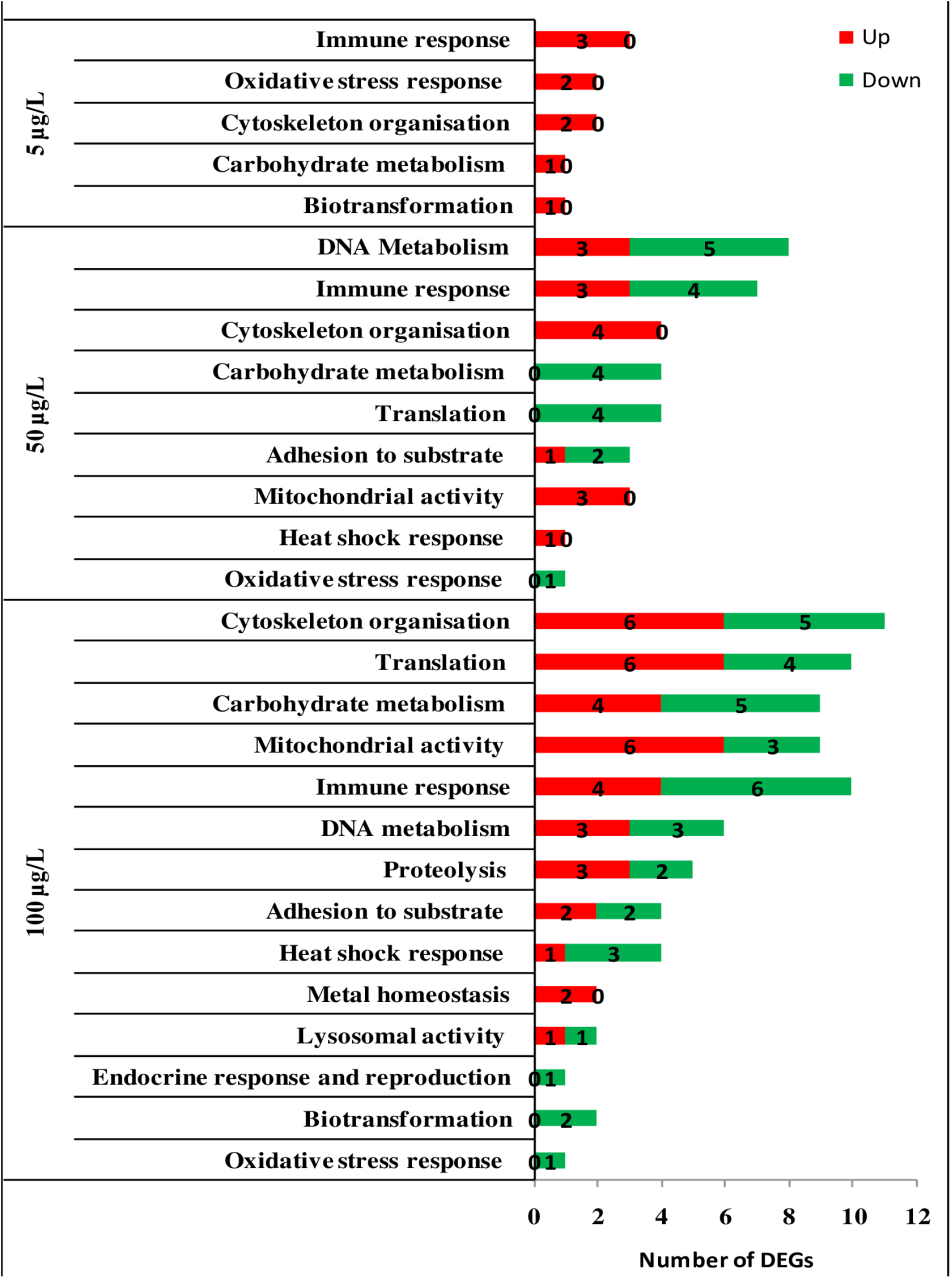
Over-representation analysis of DEGs in the digestive gland tissue of mussels exposed to B[a]P (5μg/L, 50μg/L and 100 μg/L). Showed are: experimental condition; biological processes; Number of up-regulated genes; Number of down-regulated genes. The over-represented biological processes in B[a]P-exposed animals *versus* control. (S4 Table).

Dual colour hybridization revealed evident alterations in gene expression patterns in the investigated tissues due to the B[a]P treatments (Table 2, Fig 1 and S3 Table). Our data revealed distinct patterns for 205 and 109 differentially expressed genes (DEGs) in the gills and DG, respectively. Of the 205 DEGs in gills, only 14 genes were shared among the three experimental condition datasets, whereas no target was shared between the three conditions in DG tissues (Fig 2 and S3 Table).As our new platform is based on annotated genes that are already ranked into well-known biological processes (S2 Table), thus it was easier to generate the list of biological processes involved in the mussel’s response to B[a]P-induced stress from the DEGs obtained for each condition (Figs 3 and 4).

**Table 2:** Number of DEGS depicted in animals exposed to three BaP concentrations (5, 50 and 100 μg/L) against controls (DMSO). Shown are total DEGs, number of up-regulated and Down-regulated DEGs. (4 biological replicates were considered).

	Gills			Digestive Gland		
	5 μg/L	50 μg/L	100 μg/L	5 μg/L	50 μg/L	100 μg/L
**Total DEGs**	19	138	146	9	35	80
**Up regulated**	7	102	90	9	15	35
**Down regulated**	12	36	56	0	20	45

Our data highlighted 13 contributing biological processes in gills in response to B[a]P concentrations. The response was largely dominated by the down-regulation of translation, the immune response, mitochondrial activities, and cytoskeleton-related genes in both 50 and 100 μg/L exposures (Fig 3, S3 Table). In mussels exposed to 5 μg/L B[a]P, only seven biological processes, represented by a maximum of five targets (translation), were depicted. Among the 15 biological processes used in the STREM, only “metal resistance” was not involved in the response to B[a]P challenge at the three investigated concentrations.

The main contributing biological processes in the mussel’s response to B[a]P in DG are reported in Fig 4 (S4 Table). Our data indicate that cytoskeleton, translation, mitochondrial activities, carbohydrate metabolism, immune response, and DNA metabolism (over 14 biological processes) are the most dominating processes in response to increasing concentrations of B[a]P. Furthermore, the lowest B[a]P concentration rendered only six biological processes, largely characterized by the up-regulation of the immune response, oxidative stress, and cytoskeleton-related genes and to lesser extend DNA metabolism.

### qRT-PCR

We carried out qRT-PCR to confirm and refine the relative expression levels of 10 homologous genes belonging to the most important biological processes, including the genes encoding caspase, p53, DNA ligase and topoisomerase (DNA metabolism), tubulin (cytoskeleton), hsp70, hsp27, (heat shock response), catalase, Zn-Cu superoxide-dismutase (oxidative stress), and the gst(phase II detoxification). Gill tissue was used for qRT-PCR analysis because it exhibited the most important responses to B[a]P and stronger bioaccumulated B[a]P than DG tissue (Table 1). Microarray and qRT-PCR data indicated a positive relationship in all cases (S3 Fig).

### Biomarkers of genotoxicity

#### DNA damage using the comet assay

**Fig 5:**
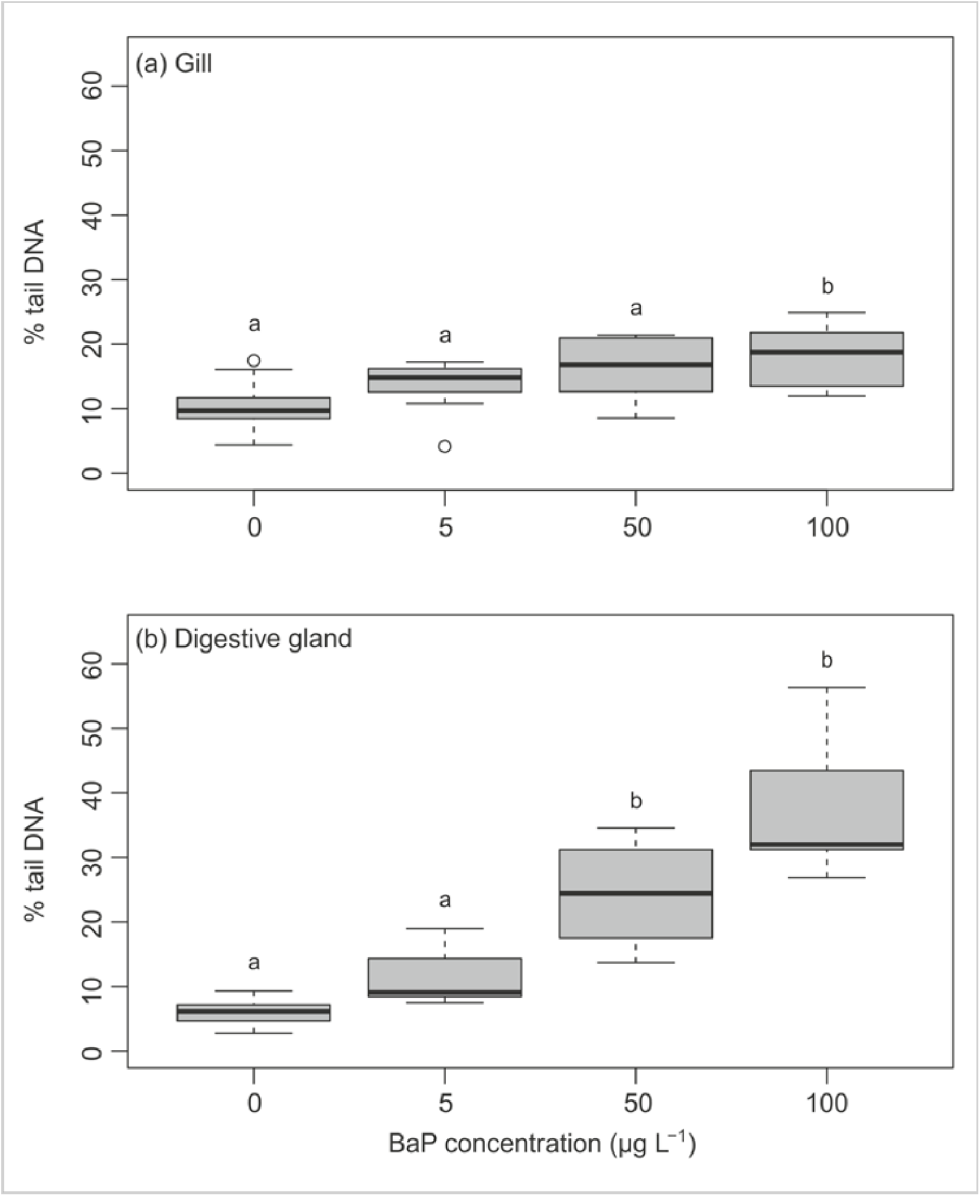
DNA strand breaks, as measured by the comet assay, in mussel (a) gill and (b) digestive gland cells after exposure to 0–100 μg/L BaP (0.02% DMSO; n = 9). Mismatching lowercase letters indicate differences between treatments according to Tukeys HSD applied as post hoc tests following a one-way ANOVA (p< 0.05).

The alkaline version of the comet assay was used to detect DNA damage including single- and double-strand breaks and alkali-labile (e.g. apurinic) sites. Induction of DNA damage was greater in DG than gills. In the DG, significant effects occurred at both 50 and 100 μg/L, compared to only 100 μg/L for the gills (one-way ANOVA, p<0.05) (Fig 5).

#### Bulky DNA adduct formation by ^32^P-postlabelling

**Fig 6:**
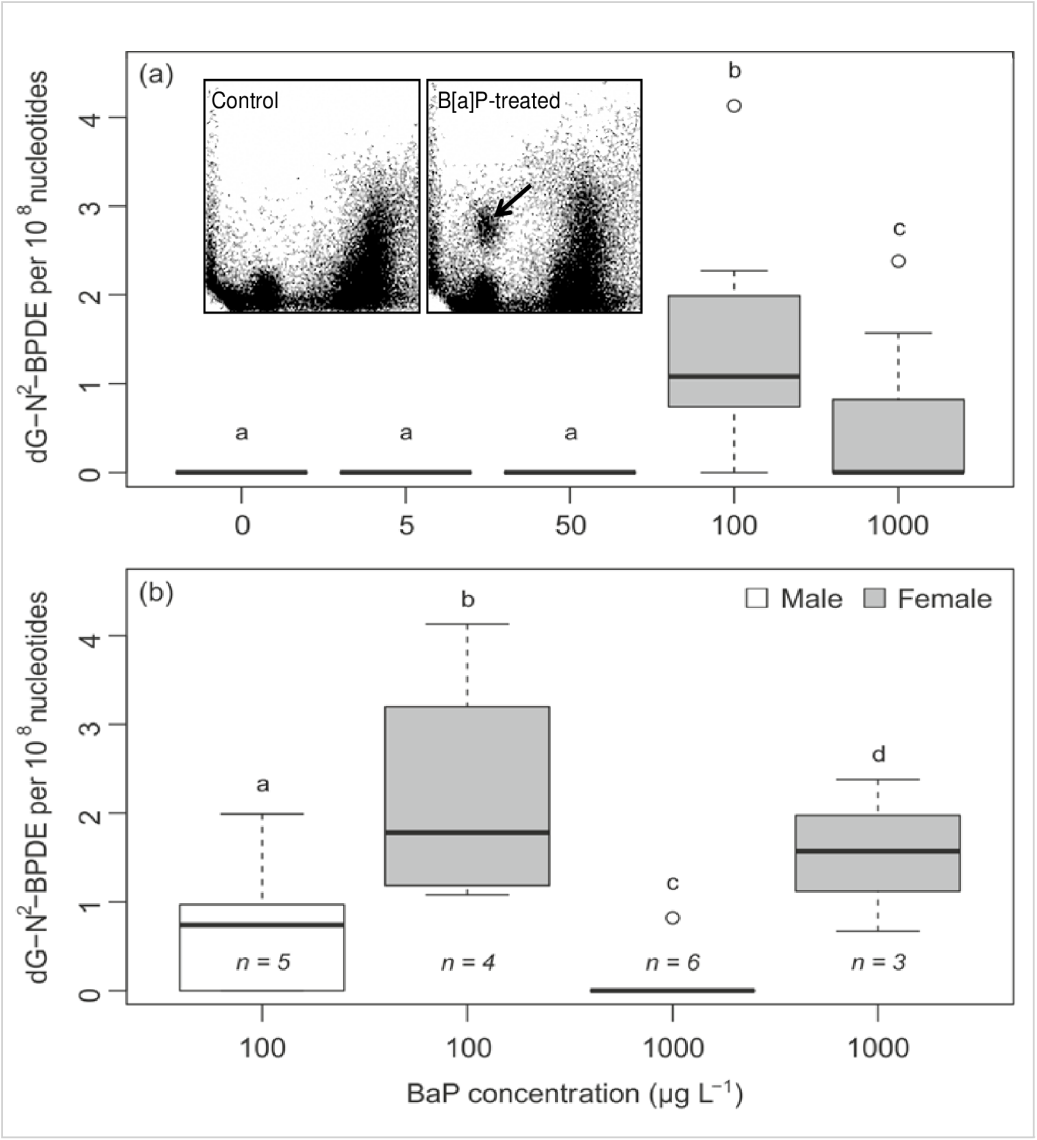
B[a]P-derived-DNA adduct levels, as measured by ^32^P-postlabelling, in mussel digestive gland cells, after exposure to 0–1000 μg/L B[a]P for 3 days. Data are shown with the sexes combined (a) and separated (for those concentrations that resulted in detectable numbers of adducts)(b). All treatments had an n of 9 in total but the distribution of sexes was not even, therefore group n values are indicated in (b). Mismatching lowercase letters indicate differences between groups according to Tukeys HSD applied as post hoc tests following a generalised linear model (p< 0.05; n = 9). Insert: Representative autoradiographic profile of DNA adducts, measured by thin-layer chromatography ^32^P-postlabelling, in digestive gland tissue exposed to B[a]P; no B[a]P-derived DNA adducts were detected in control (untreated) tissue (data not shown). The origin (OR), at the bottom left-hand corners, was cut off before exposure. The arrow indicates 10-(deoxyguanosin-*N*^2^-yl)-7,8,9-trihydroxy-7,8,9,10-tetrahydro-B[a]P (dG-*N*^2^-BPDE).

There was a distinct tissue-specific pattern in the occurrence of DNA adducts; all but one gill tissue sample had none detectable. This finding is in sharp contrast to DG tissues, 11 samples of which showed the presence of B[a]P-derived DNA adducts. On the TLC sheets the adduct pattern consisted of a single adduct spot (spot 1) (Fig 6), previously identified by mass spectrometry as dG-*N*^2^-BPDE [40]. Analysis of DNA adduct data using glm revealed significant effects of B[a]P concentration (p<0.0001) and tissue type (p<0.0001) on the level of adducts, as well as significant interactions between B[a]P concentration and tissue (p<0.05). The two higher B[a]P concentrations comprised all of the adduct-containing samples (Fig 6A).Sex-specific differences between these two concentrations (100 and 1000 μg L^-1^ B[a]P) were evident, in that female mussels showed significantly higher levels of adducts at both concentrations, although n numbers were < 5 for two groups (Fig 6B).

### Effects at tissue level

#### Lipofuscin accumulation

**Fig 7:**
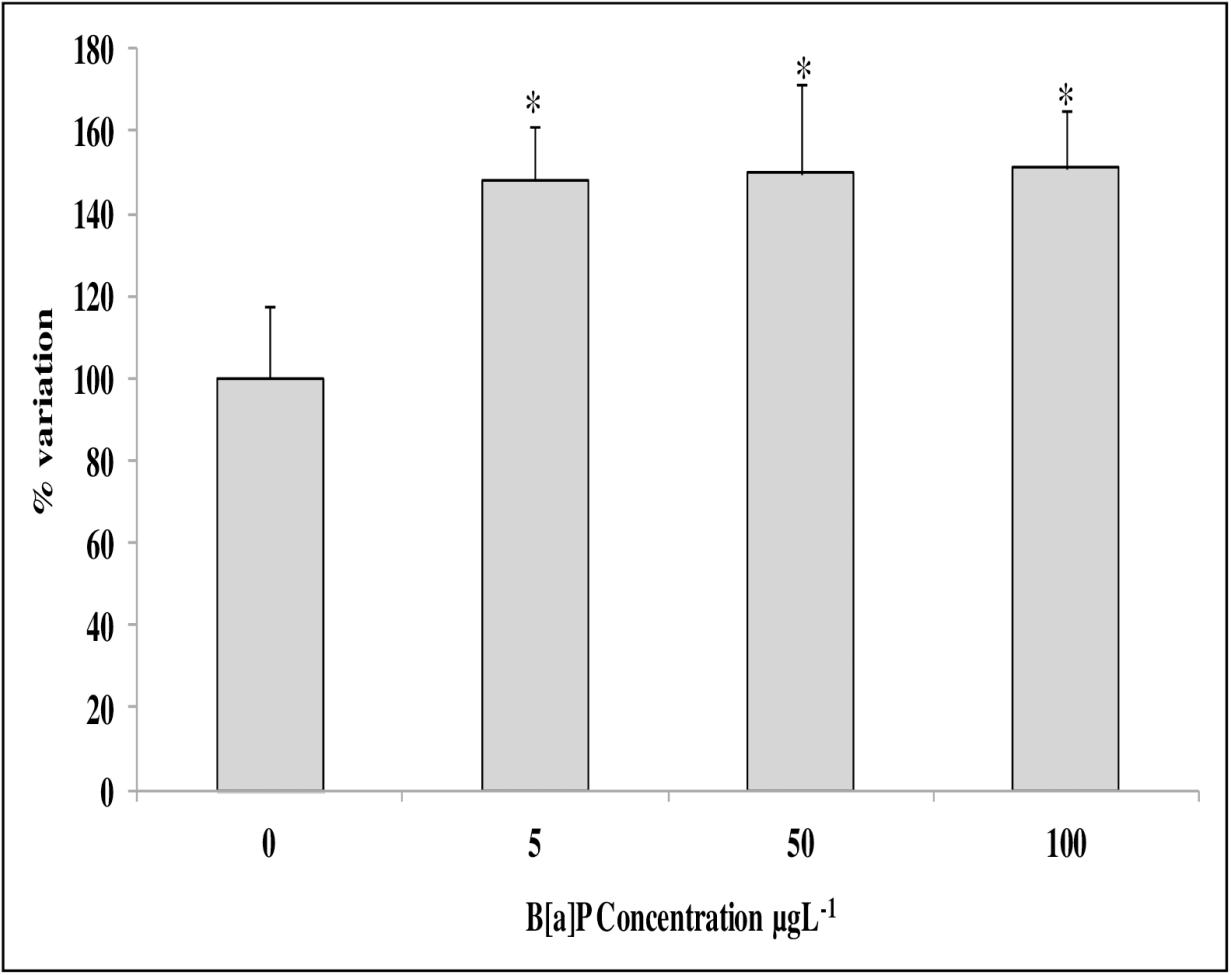
Effects of B[a]P on lipofuscin lysosomal content in the digestive gland cells of mussels, evaluated in cryostat tissue sections by the Schmorl reaction. Data, expressed as percent change with respect to control values, represent the mean ± SD of at least 10 replicates. * = p < 0.05 (Mann-Whitney *U*-test).

As shown in Fig 7, the amount of the lipofuscin accumulation in the digestive gland lysosomes of B[a]P-exposed mussels increased significantly with respect to controls, reaching a plateau already in the animals exposed to the lowest chemical concentration (5 μg/L).

#### Immunofluorescence analysis of tubulin and F-actin in digestive gland tissue sections

The consequences on cell physiology of the changes observed at transcriptomic level have been also investigated by looking at the cytoskeletal organization.

**Fig 8:**
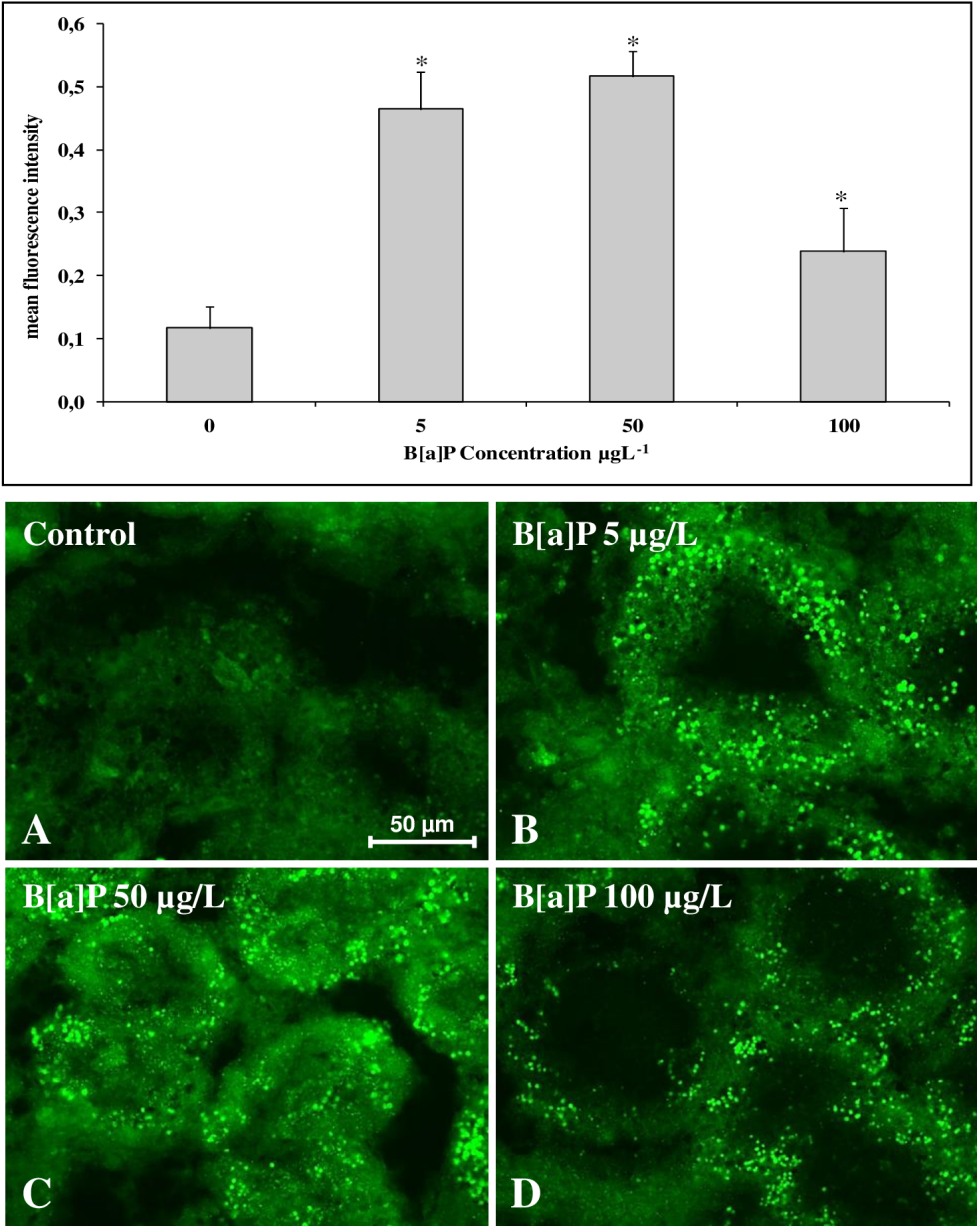
(A-D) Anti-tubulin immunohistochemical staining (Chromeo™ 488 conjugated secondary antibody) of digestive gland tissue sections from mussels exposed to different experimental conditions (A = Control; B = B[a]P 5 μg/L; C = B[a]P 50 μg/L; D = B[a]P 100 μg/L). (E) Quantitative fluorescence analysis of anti-tubulin immunoreaction. Data are mean ± SD of at least five replicates; * = p < 0.05 (Mann-Whitney *U*-test).

In particular, the possible effects on tubulin, which mRNA content increases in B[a]P-exposed mussels, and actin showing no change of the transcription of its coding gene, were evaluated using an immunohistochemical approach. The results reported in Fig 8 clearly show that the tubulin amount in the digestive gland cells increases in mussels exposed to B[a]P increasing concentrations with a bell-shape trend reaching a maximum in the animals exposed to 5–50 μg/L. In these animals, the cytoplasm of the cells contains numerous highly fluorescent granules that contribute to determine the observed tubulin increase.

**Fig 9:**
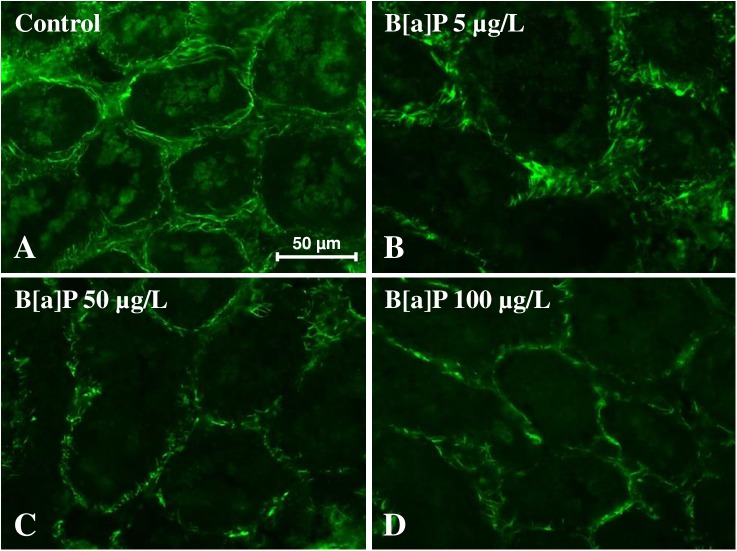
Fluorescent staining of the F-actin cytoskeleton by Green Fluorescent Phalloidin Conjugate in digestive gland sections of mussels exposed to different experimental conditions (A = Control; B = B[a]P 5 μg/L; C = B[a]P 50 μg/L; D = B[a]P 100 μg/L).

In the digestive gland cells of the mussels exposed to B[a]P, the amount of F-actin seems to be only slightly decreased but the cytoskeletal architecture is, at least in part, lost. At the higher B[a]P concentrations, actin cytoskeletal alterations are particularly evident for what concerns the cortical cell compartment and its role in the structure of the digestive gland tubule organization (Fig 9).

## Discussion

We have presented the development of a new low density oligonucleotide microarray to study alterations in mussel gene expression profiles after exposure to anubiquitous environmental stressor. Genes represented on the microarray were identified from public databases, mainly NCBI Genbank. The gene list included 465 annotated genes known to be implicated in the stress response of bivalves [3,11,4,29,2]. The annotated genes were collected in different gene data banks and represent a more complete list among genes involved in the selected pathways involved in the mussel’s stress response. Some features of the novel ‘STREM-chip’ that guarantee a good performance and reliable data include the simultaneous presence of eight replicated sub-arrays on the same slide and the use of a single probe covering the 3’ region of the mRNA, improving the accuracy of measurements. Moreover, to optimize data confidence, dual color hybridizations were performed in at least quadruplicate. For each sub-array, each target was triplicated and four biological replicates considered, resulting in 12 values for each target. In addition, the eight sub-arrays may assure at least two experimental conditions (considering four biological replicates per condition) in the same slide.

To identify DEGs, we did not set a default threshold for the fold change a priori, but instead the statistical criterion for significance was adopted, in order to appreciate moderate gene modulations. The new platform presents an advantage of being open to any additional target based on the increase in bivalve species sequencing, especially *Mytilus* sp.

To assess the potential use of the new array platform, we tested the effect of B[a]P, a relevant environmental contaminant, in two mussel tissues, the gills and DG. Chemical data suggested that the concentration of B[a]P was higher in the gills than the DG. The B[a]P concentration in the DG after exposure to 50 μg/L B[a]P was of a similar order of magnitude as in [24] after exposure to 19 μg/L/animal (10.29 μg/g). Such levels are also consistent with the whole body burden of total polycyclic aromatic hydrocarbons (PAHs) in field sampled mussels from the Irish Sea (0.13–22.48 μg/g) [44], suggesting that they are environmentally relevant.

Interesting information could be gleaned from the transcriptomic data. As expected, we observed a marked regulation of genes involved in DNA metabolism in both tissues at B[a]P concentrations of 50 and 100 μg/L. Our data suggest the occurrence of a DNA repair or apoptosis event with respect to the applied B[a]P concentration. Investigation of DNA damage response genes coding for caspase (HQ424451.1) revealed a marked up-regulation of apoptotic genes in mussels exposed to 50 or 100 μg/L B[a]P. However, DNA repair-related enzymes (DNA ligase:AJ624686.1; p53:KC545827.1) were markedly up-regulated in animals exposed to 5 μg/L B[a]P (data confirmed by qRT-PCR), except for topoisomerase, which was down-regulated (AF227976.1).

Mussel’s cells may respond to DNA damage via p53-mediated cell cycle arrest or programmed cell death [45]. The p53 family of proteins has been demonstrated to be involved in molluscan leukemic hemocytes, and to be inactivated by sequestration in the cytoplasm [46,47]. Furthermore, bulky B[a]P-DNA adducts (i.e. dG-*N*^2^-BPDE) are reported to be removed by nucleotide excision repair (NER) enzymes [20], including DNA ligase [48]. NER is dependent on the base pair conformation, suggesting a direct implication of DNA ligase-related proteins in such processes. Finally, topoisomerase, a DNA replication/repair-related enzyme has been reported to be highly active in mammalian cells exposed to PAHs [49]. PAH-DNA adducts alter the execution or fidelity of enzymatic DNA transcription and may cause mutations and cancer [50,20]. The dissimilar data obtained in this work for topoisomerase may be explained by the higher replication rate of mammalian cells maintained in culture conditions [50] compared to *in vivo*-sampled cells, as well as the difference in concentrations used in our study compared to the exposure conditions for model mammalian cells.

Investigations of DNA damage (comet assay) and DNA adduct formation (^32^P-postlabelling) showed higher genotoxicity in mussels exposed to 50 and 100 μg/L B[a]P, which is in line with the gene expression data. Moreover, our results suggest a higher genotoxicity in DG cells compared to gill cells. Previous work on the location and concentration of mono-oxygenase enzymes required for the transformation of B[a]P into its reactive intermediates in mussels, indicates much higher concentrations in DG [51], which supports our finding of higher % tail DNA values in this tissue compared to the gills, despite less B[a]P accumulation. Oxidative DNA lesions (i.e., the formation of 8-oxo-dG) have also been found to be more prevalent in DG cells than the gills after B[a]P exposure [21,24]. Livingstone et al. [51] reported cytochrome P450 enzyme activity in the DG only and the highest activities of P450-related enzymes (including B[a]P hydoxylase) in this organ (i.e.DG). This is consistent with higher levels of B[a]P-derived DNA adducts (i.e. dG-*N*^2^-BPDE) in this tissue / organ reported here. The cytochrome P450 monooxygenase system also provides a potential explanation for the sex-specific difference in BPDE-adducts that we observed, as the activities of these enzymes were also higher in females than males, implying that more of the genotoxic intermediates may be present in their digestive gland [51].

Another marked response of mussels to increasing concentrations of B[a]P in gills is the down-regulation of genes related to the translation process. Thirty-five of 38 DEGs and 31 of 36 DEGs were down-regulated in mussels exposed to 50 and 100 μg/L B[a]P, respectively, though the interpretation of this may be complex because these genes also encode proteins that are implicated in multiple cellular pathways. Of these down-regulated DEGs, 30 and 38 encode ribosomal protein subunits (S4 Table) in mussels exposed to 50 and 100 μg/L B[a]P, respectively. This down-regulation may indicate that mRNA-directed protein synthesis is reduced in mussels exposed to higher B[a]P loads. PAHs are known to interfere with protein synthesis, as the abundance of proteins involved in protein synthesis and degradation, ATP supply and structural proteins changes in response to B[a]P in *M*. *Galloprovincialis*[26]. Ribosome biogenesis has been reported to be affected by environmental stressors in bacteria [52]. In this context, it has also been suggested that both transcription and translation are regulated in zebrafish in response to environmental contaminants [53].

In this study, mitochondrial activities and carbohydrate metabolism are reported to be among the processes contributing to the mussel’s response to B[a]P exposure in two important target tissues. Recently, it has been reported that the regulation of ATP synthase F0 subunit 6 in mussels exposed to 10 μg/L B[a]P using a proteomics approach [26]. The regulation of ATP concentration is linked to inner mitochondrial membrane, through which the respiration process supplies energy to cells. Similarly, proteomic analysis of freshwater mussel *D*. *polymorpha* exposed to B[a]P for 7 days identified proteins associated with cellular metabolism (N-acetyltransferase 8-like and probable aspartate aminotransferase) [54].

B[a]P exposure can be considered as a potent inducer of oxidative stress in mussel tissues [25,55,26,56]. The production of oxy-radicals in mussels has been reported to be mediated by a higher range of contaminants, including B[a]P[57,58]. In this work, even though the number of DEGs involved in the oxidative stress response present in the new platform (i.e., 10) was less than other marked processes (e.g. translation or immune response), the results indicate clear effects as 80% of the genes involved in the oxidative stress response were affected in the gills and DG of mussels exposed to higher B[a]P concentrations. In this case, the observed transcriptomic changes were indicative of oxidative damage in the cells, and the lipofuscin lysosome content was significantly increased.

In mussels exposed to higher B[a]P concentrations, the heat shock response was also depicted as a contributing process in the gill and DG tissues. Among the stress proteins, the heat shock proteins are molecular chaperones involved in the assembly, folding, and intracellular transport of proteins, protecting against stress-associated cellular damage [59].

Among the genes present in our microarray that code for protein folding are calreticulin and FK506-binding protein. Calreticulin is known to bind mis-folded proteins, preventing their export from the endoplasmic reticulum to the Golgi apparatus [60]. Small heat shock proteins, such as HSP27, HSP26, and FK506-binding protein, are likely to act as chaperones for the maintenance of cytoskeletal structural elements during stress [61]. Calreticulin and FK506-binding protein were recently reported to be highly up-regulated in *M*. *galloprovincialis* with respect to *M*. *trossulus* exposed to higher heat stress. In addition, oxidative stress may induce heat shock protein phosphorylation, resulting in actin binding and the induction of apoptosis [62]. Thus, chaperone induction may represent an effort to protect the cells against the stress effect caused by impaired cellular responses associated with transcriptional regulation.

Exposure to PAHs has a number of effects on the immune function in marine organisms. Decreased lymphocyte proliferation has been observed in Japanese medaka (*Oryziaslatipes*) [63] and bluegill sunfish (*Lepomismacrochirus*) following dietary exposure to three different PAHs [64]. Altered *in vitro* proliferation of leukocytes from common carp has been reported after exposure to 3-methylcholanthrene. In our case, the immune response process was affected at the transcriptional level in response to B[a]P exposure in the two investigated tissues. This may attempt to speculate a possible effect ofB[a]P also in cells involved in immune response which are always present in the investigated tissues. The down-regulation trend was most dominant in the DEGs involved in immune response, suggesting a possible immune-toxicity of the applied concentrations. The study of the B[a]P effect on the immune cells could contribute to clarify this aspect.

The transcriptional response of mussels exposed to increasing concentrations of B[a]P was also marked by the down-regulation of genes encoding proteins involved in cytoskeleton maintenance and repair (Figs 3 and 4). Cytoskeletal defence was previously proposed as a potential mechanism for increased stressors, such as thermo-tolerance in mussels [6]. Increased production of reactive oxygen species (ROS) has been reported to severely affect the actin cytoskeleton in mussel hemocytes following exposure to B[a]P[56]. Recently, it has also been induction of α-tubulin in B[a]P-exposed mussels[27]. This is a key cytoskeletal component responsible for microtubule polymerization, cell transport, and motility. The study therefore reinforces the influence of all PAHs in cytoskeleton organization[27].

In the present study, tubulin appears to be one of the few cytoskeleton genes up-regulated following exposure to different concentrations of B[a]P. To complement B[a]P induced transcriptomic changes, the effects of B[a]P on the cytoskeleton structure of the digestive gland cells was also investigated. The increase in the tubulin mRNA content showed an enhancement of this protein in the cytoplasm of the digestive gland cells. Using an immuno-histochemical approach, it was possible to verify that in B[a]P exposed mussels, it induces only limited effects on the tubulin filament structure but a large amount of tubulin was found to be sequestered in cytoplasmic granules. The formation of granules containing tubulin has been previously described in mammalian and molluscan cells due to different stress/pathological conditions [65,43]. It could therefore hypothesized that B[a]P affect tubulin filaments via different mechanisms(e.g. by inducing oxidative stress) with the sequestration of the damaged tubulin monomers and part of the filaments in the granules. The decrease of free tubulin monomers should stimulate the tubulin neosynthesis [66] through the enhancement of the protein gene transcription in an attempt to maintain in the cells the cytoskeleton architecture needed for important cell functions such as vesicles transport and autophagic activity.

We did not observe any change on the actin mRNA levels in B[a]P exposed mussels. This indicates the absence of effects of the chemical on the actin cytoskeleton. In contrast, the immuno-fluorescence analysis of F-actin clearly demonstrated a relevant alteration of the actin structure in the digestive glands of treated mussels. The noxious effects of B[a]P on the F-actin architecture have been previously reported by various authors in mammalian cells [67] and in mussel’s haemocytes [56]. In this last case, the B[a]P effects were mainly related to the cortical component of the actin cytoskeleton structures. Our results demonstrated that B[a]P affects the actin ring in the cortex area involved in cell-cell contacts. These effects, which are particularly relevant at the higher B[a]P concentrations (50–100 μg/L), also imply an alteration of the organization of the digestive gland tubules.

It should be noted that the exposure to H_2_O_2_ of mammalian monolayer intestinal cells showed similar effects on the actin cytoskeleton with the loss of the actin structure present in the cortex area [68]. In our study, mussel exposed to B[a]P induced oxidative stress as demonstrated by the significant increase of lysosomal lipofuscin accumulation and by the activation of the transcription of genes related to the antioxidant responses (e.g.catalase and SOD). It therefore seems possible that oxidative stress may represent an important component in the mechanism of action of B[a]Pin mussels, at least for what concerns the observed negative effects on the cytoskeletal structure of the digestive gland cells.

In the present study, adhesion to substrate was modulated at the transcriptomic level in both tissues upon exposure to B[a]P. Similar results were observed from proteomic characterization of the DG from *M*. *galloprovincialis* after exposure to sub-lethalB[a]P concentrations. The down-regulation of this process by organic compounds could lead to impairment of the force-maintaining structures during the catch state, compromising the adhesion (byssus strength) and motility processes in exposed mussels. Investigation of this parameter would provide a link between transcriptomic studies and organism responses which could be extrapolated to population dynamics studies.

Finally, it is important to note that transcriptomic data may provide an overview of the cell’s response to stress in term of changes in the genes products. Changes in the mRNA profiles may be related to the maintenance of cell/tissue homeostasis. In this case, the organism appears to be healthy but has a decreased capacity to adapt to further environmental changes. However, alteration of the mRNA profile could also indicate that the organisms are not more capable of coping with the toxic environment [69]. To clarify these different situations, it is important to associate the transcriptomic data with the results of functional studies of cells/tissues related to different stress response pathways. One advantage of the new platform is that each biological process represented in the array could be related to the alteration of a particular biological function using previously developed biomarkers. In this work, gene expression changes were related to cytoskeleton organization (tubulin immuno-histochemistry), oxidative stress (lipofuscin content), and innovative studies on DNA damage and autophagy regulation (Sforzini et al., unpublished data).

## Conclusions

In conclusion, we have demonstrated that the novel low density DNA microarray is a robust and valid tool for obtaining insights of the molecular responses of *Mytilussp*., a model organism widely used in ecotoxicological studies. The new platform allows easy retrieval of the involved biological processes in response to stress because targets are already clustered according to their biological implications. The new platform remains open to eventual implementation of any new targets or processes in view of the full genome sequencing of *Mytilus* sp. Moreover, the presence of eight sub-arrays per platform is associated with a reduction in the cost/experimental effort and will allow future routine use of this microarray in field or biomonitoring studies. The integration of transcriptomic data with the effects on cell/tissue functions as determined by other novel sub-lethal biomarker responses provide a more biologically meaningful interpretation of the physiological alteration, e.g. rearrangement of nucleus activity on the capability of the mussels to adapt to toxic environments. In particular, despite an increase in activity of the genes coding for proteins involved in DNA repair, DNA damage was observed. The changes in DEGs coding for cytoskeleton proteins reflect an alteration in its structure in DG cells and the increase in mRNA coding for antioxidant enzymes is not sufficient to protect the cells from oxidative stress.

## Supporting information

S1 Fig Concentration of benzo[a]pyrene (B[a]P) in seawater over the first 24 h of 3 d exposure at 0–100 μg L^-1^. Data are means ± SD (n = 3). Dashed lines represent the nominal concentrations at 5, 50 and 100 μg L^-1^. At 24h, B[a]P concentrations in the 5 μg L^-1^ group were below the limit of detection (0.25 μg L-1). Although a 1000 μg L^-1^ exposure was also performed (for DNA adduct analysis only), water concentrations were not measured for this treatment for logistical reasons.
(DOCX)

S2 Fig A typical dual color hybridization analysis of Cy3/Cy5-labelled cDNAs from B[a]P-treated vs control mussels, obtained by means of a dual laser source microchip scanner. For data normalization, the two channels were balanced on RNA intensities.
(DOCX)

S3 Fig qRT-PCR confirmation of microarray data. Targets expressions have been analyzed by real-time PCR, using a 18S rRNA, Beta actin and Ribol27 as reference genes for data normalization. Microarray data for the investigated genes were confirmed. These results indicate a high accuracy and sensitivity of the STREM-Ship that was able to detect even small change of expression. Data represent the mean of at least four independent experiments. Calculation of relative expression levels and statistics (pairwise randomization test, p < 0.05) were obtained using the REST software[37]. Experimental coefficient of variation (CV) was below 5% for all the investigated targets.
(DOCX)

S1 Table qRT-PCR primers and Taqman probes. (DOCX)

S2 Table The total array sequence names. Shown is the description and the associated Biological processes (Additional information to Figs 1,2,3 and 4).
(XLSX)

S3 Table M-Values of the 205 DEGs in gills tissues in at least one condition during the exposure to B[a]P concentrations (without B[a]P supply is considered as the reference). Additional information to Figs 1 and 3.
(XLSX)

S4 Table M-Values of the 109 DEGs indigestive gland tissues in at least one condition during the exposure to B[a]P concentrations (without B[a]P supply is considered as the reference). Additional information to Figs 2 and 4.
(XLSX)

## References

[1] Tanguy A, Bierne N, Saavedra Pina B, Bachère E et al (2008) Increasing genomic information in bivalves through new EST collections in four species: development of new genetic markers for environmental studies and genome evolution. Gene 31;408(1–2):27–36. doi: 10.1016/j.gene.2007.10.021 18054177 10.1016/j.gene.2007.10.021

[2] Lockwood BL, Sanders JG, Somero GN (2010) Transcriptomic responses to heat stress in invasive and native blue mussels (genus Mytilus): molecular correlates of invasive success. J Exp Biol 213: 3548–58. doi: 10.1242/jeb.046094 20889835 10.1242/jeb.046094

[3] Banni M, Negri A, Mignone F, Boussetta H, Viarengo A et al (2011) Gene expression rhythms in the mussel Mytilusgalloprovincialis (Lam.) across an annual cycle.PLoSOne 6 (5), e18904. 10.1371/journal.pone.0018904 PMC3088662 21573210

[4] Canesi L, Negri A, Barmo C, Banni M, Gallo G et al (2011) The organophosphate chlorpyrifos interferes with the responses to 17b-estradiol in the digestive gland of the marine mussel Mytilusgalloprovinciali. PLos One 6 (5),e19803 doi: 10.1371/journal.pone.0019803 21625485 10.1371/journal.pone.0019803 PMC3098840

[5] Negri A, Oliveri C, Sforzini S, Mignione F, Viarengo A et al (2013) Transcriptional response of the mussel Mytilusgalloprovincialis (Lam.) following exposure to heat stress and copper. PLoS One. 2013; 8(6), e66802 doi: 10.1371/journal.pone.0066802 23825565 10.1371/journal.pone.0066802 PMC3692493

[6] Dalziel AC, Rogers SM, Schulte PM (2009) Linking genotypes to phenotypes and fitness: how mechanistic biology can inform molecular ecology. MolEcol 18(24):4997–5017. 10.1111/j.1365-294X.2009.04427.x 19912534

[7] Matocq MD (2009) A microarray's view of life in the desert: adding a powerful evolutionary genomics tool to the packrat's midden. 2009. MolEcol 18(11):2310–2. 10.1111/j.1365-294X.2009.04172.x 19389176

[8] Bayne BL (1976) Marine Mussels: Their Ecology and Physiology Cambridge University Press, London, UK (1976)

[9] Banni M, Jebali J, Daubeze M, Clerandau C, Guerbej H et al (2005) Monitoring pollution in Tunisian coasts: application of a classification scale based on biochemical markers. Biomarkers. 10(2–3):105–16. doi: 10.1080/13547500500107497 16076726 10.1080/13547500500107497

[10] Attig H, Dagnino A, Negri A, Jebali J, Boussetta H et al (2010) Uptake and biochemical responses of mussels Mytilusgalloprovincialis exposed to sublethal nickel concentrations. 2010. Ecotoxicol Environ Saf 73(7):1712–9. doi: 10.1016/j.ecoenv.2010.08.007 20800282 10.1016/j.ecoenv.2010.08.007

[11] Gracey AY, Chaney ML, Boomhower JP, Tyburczy WR, Connor K, et al (2008) Rhythms of Gene Expression in a Fluctuating Intertidal Environment. Current Biol 18: 1501–1507. 10.1016/j.cub.2008.08.049 18848447

[12] Di Y, Schroeder D. C, Highfield A, Readman J.W, Jha AN (2011) Tissue-specific expression of p53 and ras genes in response to the environmental genotoxicant benzo(a)pyrene in marine mussels. Environ SciTechnol 45, 8974–8981. 10.1021/es201547x 21899289

[13] Di Y, Aminot Y, Schroeder DC, Readman JW, Jha AN (2017) Integrated biological responses and tissue-specific expression of p53 and ras genes in marine mussels following exposure to benzo(α)pyrene and C60 fullerenes, either alone or in combination. Mutagenesis 32, 77–90. doi: 10.1093/mutage/gew049 28011749 10.1093/mutage/gew049

[14] Banni M, Bouraoui Z, Clerandeau C, Narbonne J F, Boussetta H (2009a) Mixture toxicity assessment of cadmium and benzo a pyrene in the sea worm hedistediversicolor. Chemosphere 77 (7), 902–906. 19758679 10.1016/j.chemosphere.2009.08.041

[15] Telli-Karakoç F, Ruddock PJ, Bird DJ, Hewer A, Van Schanke A et al (2002) Correlative changes in metabolism and DNA damage in turbot (Scophthalmus maximus) exposed to benzo[a]pyrene. Mar Environ Res. 54(3–5):511–5. 12408610 10.1016/s0141-1136(02)00192-7

[16] Jha AN, Hutchinson TH, Mackay JM, Elliott BM, Dixon DR (1996) Development of an in vivo genotoxicity assay using the marine worm Platynereisdumerilii (polychaeta: Nereidae). Mut Res 359 (2), 141–150. 8598832 10.1016/s0165-1161(96)90260-5

[17] Wessel N, Rousseau S, Caisey X, Quiniou F, Akcha F (2007) Investigating the relationship between embryotoxic and genotoxic effects of benzo alpha pyrene, 17 alpha-ethinylestradiol and endosulfan on crassostreagigas embryos. Aqua Toxicol 85 (2): 133–142. 10.1016/j.aquatox.2007.08.007 17904659

[18] Sogbanmu TO, Nagy E, Phillips DH, Arlt VM, Otitoloju AA et al (2016) Lagos lagoon sediment organic extracts and polycyclic aromatic hydrocarbons induce embryotoxic, teratogenic and genotoxic effects in Danio rerio (zebrafish) embryos. Environ SciPollut Res Int. 23(14):14489–501. 10.1007/s11356-016-6490-y PMC4943991 27068906

[19] Kucab JE, van Steeg H, Luijten M, Schmeiser HH, White PA et al (2015) TP53 mutations induced by benzo[a]pyrene-7,8-diol-9,10-epoxide in Xpa-WT and Xpa-Null Human TP53 Knock-In (Hupki) mouse embryo fibroblasts. Mutat Res–Fundamental and Molecular Mechanisms of Mutagenesis 773: 48–62. 10.1016/j.mrfmmm.2015.01.013 PMC4547099 25847421

[20] Long AS, Lemieux CL, Arlt VM, White PA (2016) Tissue-specific in vivo genetic toxicity of nine polycyclic aromatic hydrocarbons assessed using the Muta™Mouse transgenic rodent assay. ToxicolAppPharmacol 290: 31–42. 10.1016/j.taap.2015.11.010 PMC4712826 26603514

[21] Canova S, Degan P, Peters LD, Livingstone DR, Voltan R, Venier P.(1998). Tissue dose, DNAadducts, oxidative DNA damage and CYP1A-immunopositive proteins in mussels exposed to waterborne benzo[a]pyrene. Mutat Res-Fundamental and Molecular Mechanisms of Mutagenesis 399 (1): 17–30. 10.1016/s0027-5107(97)00263-7 9635486

[22] Ling H, Sayer J M, Plosky BS, Yagi H, Boudsocq F et al (2004) Crystal structure of a benzo[a]pyrene diol epoxide adduct in a ternary complex with a dna polymerase. PNAS 101 (8): 2265–2269. doi: 10.1073/pnas.0308332100 14982998 10.1073/pnas.0308332100 PMC356939

[23] Banni M, Negri A, Dagnino A, Jebali J, Ameur S et al (2010) Acute effects of benzo[a]pyrene on digestive gland enzymatic biomarkers and dna damage on mussel mytilusgalloprovincialis. Ecotoxicol and Environ Saf 73 (5): 842–848. 10.1016/j.ecoenv.2009.12.032 20071027

[24] Akcha F, Burgeot T, Budzinski H, Pfohl-Leszkowicz A, Narbonne J-F (2000) Induction and elimination of bulky benzo[a]pyrene-related dna adducts and 8-oxo-d-guo in mussels mytilusgalloprovincialis exposed in vivo to bB[a]p P contaminated feed. Mar EcolProgSer 205: 195–206.

[25] Tian S, Pan L, Sun X (2013) An investigation of endocrine disrupting effects and toxic mechanisms modulated by benzo[a]pyrene in female scallop Chlamysfarreri. AquatToxicol.15(144–145):162–71. 10.1016/j.aquatox.2013.09.031 24185101

[26] Maria V L, Gomes T, Barreira L, Bebianno M J (2013) Impact of benzo(a)pyrene, cu and their mixture on the proteomic response of mytilusgalloprovincialis. AquatToxicol 144-(145): 284–95. 10.1016/j.aquatox.2013.10.009 24211336

[27] Farkas J, Bergum S, Nilsen EW, Olsen AJ, Salaberria I et al (2015) The impact of tio2 nanoparticles on uptake and toxicity of benzo(a)pyrene in the blue mussel (mytilus edulis). STOTEN 511 (0): 469–476. 10.1016/j.scitotenv.2014.12.084 25574974

[28] Altenburger R., Scholz S., Schmitt-Jansen M., Busch W., Eschert B. I., 2012 Mixture toxicity revisited from a toxicogenomic perspective. Environ SciTechnol 46 (5): 2508–2522. 10.1021/es2038036 22283441

[29] Venier P, De Pittà C, Pallavicini A, Marsano F, Varotto F et al (2006) Development of mussel mRNA profiling: Can gene expression trends reveal coastal water pollution? Mutat Res, 602, 121–134. doi: 10.1016/j.mrfmmm.2006.08.007 17010391 10.1016/j.mrfmmm.2006.08.007

[30] Dondero F, Banni M, Negri A, Boatti L, Dagnino A, et al (2011) Interactions of a pesticide/heavy metal mixture in marine bivalves: a transcriptomic assessment. BMC Genomics 12: 195 doi: 10.1186/1471-2164-12-195 21496282 10.1186/1471-2164-12-195 PMC3094310

[31] D’Agata A, Fasulo S, Dallas L J, Fisher AS, Maisano M, Readman JW, Jha AN (2014) Enhanced toxicity of bulky titanium dioxide compared to ‘fresh’ and ‘aged’ nano-TiO2 in marine mussels (Mytilusgalloprovincialis). Nanotoxicology 8, 549–558. doi: 10.3109/17435390.2013.807446 23697396 10.3109/17435390.2013.807446

[32] Bignell JP, Stentiford GD, Taylor N GH, Lyons BP (2011) Histopathology of mussels (Mytilus sp.) from the Tamar Estuary, UK. Mar Environ Res 72: 25–32. doi: 10.1016/j.marenvres.2011.05.004 21703678 10.1016/j.marenvres.2011.05.004

[33] Dallas LJ, Bean TP, Turner A, Lyons BP, Jha AN (2013) Oxidative DNA damage may not mediate Ni-induced genotoxicity in marine mussels: Assessment of genotoxic biomarkers and transcriptional responses of key stress genes. Mutat Res/Genetic Toxicology and Environmental Mutagenesis 754: 22–31. 10.1016/j.mrgentox.2013.03.009 23591161

[34] Devier M-H, Augagneur S, Budzinski H, Le Menach K, Mora P, Narbonne J-F et al (2005) One-year monitoring survey of organic compounds (PAHs, PCBs, TBT), heavy metals and biomarkers in blue mussels from the Arcachon Bay, France.J Enviro Monit. 5;7:224. 10.1039/b409577d 15735781

[35] Chomczynski P, Sacchi N (1987) Single-step method of RNA isolation by acid guanidinium thiocyanate-phenol-chloroform. extraction. AnnalBioche 162: 156–169. 10.1006/abio.1987.9999 2440339

[36] Conesa A, Götz S, García-Gómez JM, Terol J, Talón M, et al (2005) Blast2GO: A universal tool for annotation, visualization and analysis in functional genomics research. Bioinformatics 21: 3674–3686. doi: 10.1093/bioinformatics/bti610 16081474 10.1093/bioinformatics/bti610

[37] Pfaffl MW, Horgan GW, Dempfle L (2002) Relative expression software tool (REST) for groupwise comparison and statistical analysis of relative expression results in real-time PCR. Nucleic Acids Res 30:e36 11972351 10.1093/nar/30.9.e36 PMC113859

[38] Al-Subiai SN, Arlt VM, Frickers PE, Readman JW, Stolpe B, Lead JR, Moody AJ, Jha AN(2012) Merging nano-genotoxicology with eco-genotoxicology: An integrated approach to determine interactive genotoxic and sub-lethal toxic effects of C-60 fullerenes and fluoranthene in marine mussels, Mytilus sp. Mutat Res-Genetic Toxicology and Environmental Mutagenesis 745, 92–103. 10.1016/j.mrgentox.2011.12.019 22230430

[39] Phillips DH, Arlt VM (2014) (32)P-postlabeling analysis of DNA adducts. Methods in Molecular Biology, 1105, 127–138. doi: 10.1007/978-1-62703-739-6_10 24623224 10.1007/978-1-62703-739-6_10

[40] Arlt VM, Stiborova M, Henderson CJ, Thiemann M, Frei E et al (2008) Metabolic Activation of benzo[a]pyrene in vitro by hepatic cytochrome P450 contrasts with detoxification in vivo: experiments with hepatic cytochrome P450 reductase null mice. Carcinogenesis, 29, 656– doi: 10.1093/carcin/bgn002 18204078 10.1093/carcin/bgn002

[41] Krais AM, Speksnijder EN, Melis JP, Indra R, Moserova M et al (2016) The impact of p53 on DNA damage and metabolic activation of the environmental carcinogen benzo[a]pyrene: effects in Trp53(+/+), Trp53(+/-) and Trp53(-/-) mice. Arch Toxicol 90:, 839–851. doi: 10.1007/s00204-015-1531-8 25995008 10.1007/s00204-015-1531-8 PMC4785204

[42] Pearse AGE (1985) Histochemistry:TheoreticalandApplied.Vol.2 ChurchillLi- vingstone, Edinburgh,pp.441–1055.

[43] Moore MN (1988) Cytochemical responses of the lysosomal system and NADPH-ferrihemoprotein reductase in molluscan digestive cells to environmental and experimental exposure to xenobiotics. Mar EcolProgSer 46: 81–89.

[44] Banni M, Sforzini S, Balbi T, Corsi I, Viarengo A et al (2016) Combined effects of n-TiO2 and 2,3,7,8-TCDD in *Mytilus galloprovincialis* digestive gland: A transcriptomic and immunohistochemical study. Environ Res 145:135–44. doi: 10.1016/j.envres.2015.12.003 26687187 10.1016/j.envres.2015.12.003

[45] Widdows J, Donkin P, Staff FJ, Matthiessen P, Law R J et al (2002) Measurement of stress effects (scope for growth) and contaminant levels in mussels (mytilus edulis) collected from the irish sea. Mar Environ Res 53 (4): 327–356. 11991207 10.1016/s0141-1136(01)00120-9

[46] Banni M, Negri A, Rebelo M, Rapallo F, Boussetta H et al (2009b) Expression analysis of the molluscan p53 protein family mrna in mussels (mytilus spp.) exposed to organic contaminants. Comp BiochemPhysiol C ToxicolPharmacol 149 (3): 414–8. 10.1016/j.cbpc.2008.09.017 18973830

[47] Kelley ML, Winge P, Heaney JD, Stephens RE, Farell JH, et al (2001) Expression of homologues for p53 and p73 in the softshell clam (Mya arenaria), a naturally occurring model for human cancer. Oncogene 8;20(6):748–58. doi: 10.1038/sj.onc.1204144 11314008 10.1038/sj.onc.1204144

[48] Walker C, Böttger S, Low B (2006) Mortalin-based cytoplasmic sequestration of p53 in a non mammalian cancer model. Am J Pathol 168(5):1526–30. doi: 10.2353/ajpath.2006.050603 16651619 10.2353/ajpath.2006.050603 PMC1606587

[49] Hess MT, Gunz D, Luneva N, Geacintov NE, Naegeli H (1997) Base pair conformation-dependent excision of benzo[a]pyrene diol epoxide-guanine adducts by human nucleotide excision repair enzymes. Mol Cell Biol 17(12):7069–76. 9372938 10.1128/mcb.17.12.7069 PMC232563

[50] Yakovleva L, Handy CJ, Yagi H, Sayer JM, Jerina DM et al (2006) Intercalating polycyclic aromatic hydrocarbon-DNA adducts poison DNA religation by Vaccinia topoisomerase and act as roadblocks to digestion by exonuclease III. Biochemistry. 20;45(24):7644–53. doi: 10.1021/bi060158h 16768460 10.1021/bi060158h

[51] Jerina DM, Chadha A, Cheh AM, Schurdak ME, Wood AW et al (1991) Covalent bonding of bay-region diol epoxides to nucleic acids. AdvExp Med Biol 283:533–53. 10.1007/978-1-4684-5877-0_70 2069024

[52] Livingstone DR and Farrar SV (1984) Tissue and subcellular distribution of enzyme activities of mixed-function oxygenase and benzo [a] pyrene metabolism in the common mussel mytilus edulis. STOTEN 39 (3), 209–235.

[53] Al Refaii A and Alix JH (2009) Ribosome biogenesis is temperature-dependent and delayed in Escherichia coli lacking the chaperones DnaK or DnaJ. MolMicrobiol 71(3):748–62. 10.1111/j.1365-2958.2008.06561.x 19054328

[54] Connolly MH and Hall BK (2008) Embryonic heat shock reveals latent hsp90 translation in zebrafish (Danio rerio). Int J Dev Biol 52(1):71–9. doi: 10.1387/ijdb.062241mc 18033674 10.1387/ijdb.062241mc

[55] Riva C, Binelli A, Rusconi F, Colombo G, Pedriali A et al (2011). A proteomic study using zebra mussels (D. polymorpha) exposed to benzo(α)pyrene: the role of gender and exposure concentrations. AquatToxicol 104(1–2):14–22. 10.1016/j.aquatox.2011.03.008 21536009

[56] Gómez-Mendikute A and Cajaraville MP (2003) Comparative effects of cadmium, copper, paraquat and benzo[a]pyrene on the actin cytoskeleton and production of reactive oxygen species (ROS) in mussel haemocytes. ToxicolIn Vitro 17(5–6):539–46. 10.1016/s0887-2333(03)00093-6 14599442

[57] Kamel N, Attig H, Dagnino A, Boussetta H, Banni M (2012) Increased temperatures affect oxidative stress markers and detoxification response to benzo[a]pyrene exposure in mussel Mytilusgalloprovincialis. Arch Environ ContamToxicol 63(4):534–43. 10.1007/s00244-012-9790-3 22903631

[58] Altenburger R, Segner H, Van der Oost R (2003) Biomarkers and PAH prospects for the assessment of exposure and effects in aquatic systems. Wiley, Chichester, UK, pp. 147–171.

[59] Garcia Martinez P, Winston GW, Metash-Dickey C, O'Hara SC, Livingstone DR (1995) Nitrofurantoin-stimulated reactive oxygen species production and genotoxicity in digestive gland microsomes and cytosol of the common mussel (Mytilus edulis L.). ToxicolApplPharmacol 131(2):332–41. 10.1006/taap.1995.1076 7716774

[60] Liu T, Pan L, Cai Y, Miao J (2015) Molecular cloning and sequence analysis of heat shock proteins 70 (HSP70) and 90 (HSP90) and their expression analysis when exposed to benzo(a)pyrene in the clam Ruditapesphilippinarum. Gene. 2015 25;555(2):108–18. doi: 10.1016/j.gene.2014.10.051 25445266 10.1016/j.gene.2014.10.051

[61] Rizvi SM, Mancino L, Thammavongsa V, Cantley RL, Raghavan M (2004) A polypeptide binding conformation of calreticulin is induced by heat shock, calcium depletion, or by deletion of the C-terminal acidic region. Molecular Cell 15(6),913–23. doi: 10.1016/j.molcel.2004.09.001 15383281 10.1016/j.molcel.2004.09.001

[62] Merck KB, Groenen PJ, Voorter CE, de Haard-Hoekman WA, Horwitz J et al (1993) Structural and functional similarities of bovine alpha-crystallin and mouse small heat-shock protein. A family of chaperones. J Biol Chem. 1993 1 15;268(2):1046–52. 8093449

[63] Dalle-Donne I1, Rossi R, Milzani A, Di Simplicio P, Colombo R (2001) The actin cytoskeleton response to oxidants: from small heat shock protein phosphorylation to changes in the redox state of actin itself. Free RadicBiol Med 15;31(12):1624–32. 10.1016/s0891-5849(01)00749-3 11744337

[64] Carlson EA, Li Y, Zelikoff JT (2002) Exposure of Japanese medaka (Oryziaslatipes) to benzo[a]pyrene suppresses immune function and host resistance against bacterial challenge. AquatToxicol 56(4):289–301. 10.1016/s0166-445x(01)00223-5 11856577

[65] Connelly H and Means JC (2010) Immunomodulatory effects of dietary exposure to selected polycyclic aromatic hydrocarbons in the bluegill (Lepomismacrochirus). Int J Toxicol 29(5):532–45. doi: 10.1177/1091581810377518 20884862 10.1177/1091581810377518

[66] Martin D, Duarte M, Lepault J, Poncet D (2010) Sequestration of free tubulin molecules by the viral protein NSP2 induces microtubule depolymerization during rotavirus infection. J Virol (84): 2522–2532. 20032187 10.1128/JVI.01883-09 PMC2820929

[67] Cleveland DW (1989) Autoregulated control of tubulin synthesis in animal cells. CurrOpin Cell Biol 1: 10–14. 10.1016/s0955-0674(89)80030-4 2629858

[68] McGarry MA, Charles GD, Medrano T, Bubb MR, Grant MB (2002) Benzo(a)pyrene, but not 2,3,7,8-tetrachlorodibenzo-p-dioxin, alters cell adhesion proteins in human uterine RL95-2 cells. BiochemBiophys Res Commun 294(1):101–7. 10.1016/S0006-291X(02)00437-0 12054747

[69] Banan A, Fields JZ, Zhang Y, Keshavarzian A (2001) iNOS upregulation mediates oxidant-induced disruption of F-actin and barrier of intestinal monolayers. Am J PhysiolGastrointest Liver Physiol 280(6):G1234–46. 10.1152/ajpgi.2001.280.6.G1234 11352817

