# Supplementary material for: Assessing the impact of Benzo[a]pyrene on Marine Mussels: Application of a novel targeted low density microarray complementing classical biomarker responses
Source: PLoS One. 2017 Jun 26;12(6):e0178460. doi: 10.1371/journal.pone.0178460 (PMC5484464; doi:10.1371/journal.pone.0178460)
Supplement: S1 Fig — Data are means ± SD (n = 3). Dashed lines represent the nominal concentrations at 5, 50 and 100 μg L-1. At 24h, B[a]P concentrations in the 5 μg L-1 group were below the limit of detection (0.25 μg L-1). Although a 1000 μg L-1 exposure was also performed (for DNA adduct analysis only), water concentrations were not measured for this treatment for logistical reasons. (DOCX) [file pone.0178460.s001.docx]

Supplementary Figure S1 : Q-RT-PCR analysis of selected target. A Confirmation of micro-array data.
