# Supplementary material for: Assessing the impact of Benzo[a]pyrene on Marine Mussels: Application of a novel targeted low density microarray complementing classical biomarker responses
Source: PLoS One. 2017 Jun 26;12(6):e0178460. doi: 10.1371/journal.pone.0178460 (PMC5484464; doi:10.1371/journal.pone.0178460)
Supplement: S2 Fig — For data normalization, the two channels were balanced on RNA intensities. (DOCX) [file pone.0178460.s002.docx]

**Supplementary figure S2:** A typical dual color hybridization analysis of Cy3/Cy5-labelled cDNAs from B[a]P-treated vs control mussels, obtained by means of a dual laser source microchip scanner. For data normalization, the two channels were balanced on RNA intensities.
