# Supplementary material for: Assessing the impact of Benzo[a]pyrene on Marine Mussels: Application of a novel targeted low density microarray complementing classical biomarker responses
Source: PLoS One. 2017 Jun 26;12(6):e0178460. doi: 10.1371/journal.pone.0178460 (PMC5484464; doi:10.1371/journal.pone.0178460)
Supplement: S3 Fig — Targets expressions have been analyzed by real-time PCR, using a 18S rRNA, Beta actin and Ribol27 as reference genes for data normalization. Microarray data for the investigated genes were confirmed. These results indicate a high accuracy and sensitivity of the STREM-Ship that was able to detect even small change of expression. Data represent the mean of at least four independent experiments. Calculation of relative expression levels and statistics (pairwise randomization test, p < 0.05) were obtained using the REST software[37]. Experimental coefficient of variation (CV) was below 5% for all the investigated targets. (DOCX) [file pone.0178460.s003.docx]

**Supplementary Figure S3**: Q-PCR confirmation of microarray data. Targets expressions have been analyzed by real-time PCR, using a 18S rRNA, Beta actin and Ribol27 as reference genes for data normalization. Microarray data for the investigated genes were confirmed, using this more sophisticated technique. These results indicate a high accuracy and sensitivity of the STREM-Ship that was able to detect even very low fold change of expression. Data represent the mean of at least four independent experiments. Calculation of relative expression levels and statistics (pairwise randomization test, p < 0.05 ) were obtained using the REST software (Pfaffl et al., 2002). Experimental coefficient of variation (CV) was below 5% for all the investigated targets.
