# Supplementary material for: Assessing the impact of Benzo[a]pyrene on Marine Mussels: Application of a novel targeted low density microarray complementing classical biomarker responses
Source: PLoS One. 2017 Jun 26;12(6):e0178460. doi: 10.1371/journal.pone.0178460 (PMC5484464; doi:10.1371/journal.pone.0178460)
Supplement: S1 Table — (DOCX) [file pone.0178460.s004.docx]

Supplementary Table S1. Q-PCR primers and Taqman probes

| Gene _ID | Probe | Sense Primer | Antisense Primer |
| --- | --- | --- | --- |
| Hsp27 (AJ625244) | CAAATGTCTATAGTCAGCAATTGGTAA | TCTTACTATCCGGGCACCAA | ACACAAGCATCCAAATGCAA |
| Hsp 70 (AJ624049) | TCCACCAGCCGACCACGTAGGACA | ACGTTGGTGCTTTGTAGGTCAT | GCCATTGATTTCGGAACCACG |
| Sod(FM177867.1) | AGTCGTCACTGTCACTGTCCTCTC | TGAAAGGAGATGGTGCTGTTAC | CAAACTCGTGAACGTGGAAAC |
| Cat (AY580271.1) | ACAGCTTGTCTGCCTGCTCAGCAC | ACAAGGATGGACAGGCATACTAC | AATCACGGATGGCATAATCTGGA |
| Gst (AF527010.1) | ACGCCTGTGTCCCCAAACAAGTGG | AACTGACCACTTCAAGAATATGCC | AGAAAGTCTGCCATTTACAAAGCT |
| Casp (HQ424451.1) | CCATTCCCTTCAACCTCCTCGCCG | TGTCTTAGCGTTCTGTATCAGTAC | AGCTTTCTGCTGGGAAAATGAC |
| P53(KC545827.1) | - | AATGTCACAAGCTTCAGTTTCAAC | TAGATGTGATGTTTGTGTATCCCC |
| Topo (AF227976.1) | - | GCTTCCTTAGCTGTGCTGGT | ACACCATTTCTGGCCAAATC |
| DNA L (AJ624686.1) | - | CGACAGCCTGAATTGCAGCAG | CTGGGGCTTTCTCTCATGGTTC |
| Tubulin (AJ516796) | TGTTGCCTGCACCACTCTGTCCGA | GCCAAATCTTCAGACCAGACAAC | ACCTTCTGTGTAATGTCCCTTGG |
| RiboL27 (AJ625928) | TGCGCCATTCAGCACAAGAACTACCT | AAGCCATGGGCAAATTTATGAAAA | TTTACAATGACTGCTTTACGACCT |
| Actin (AJ625116) | ACGCCAACACCGTCTTGTCTGGTGG | GTGTGATGTCATATCCGTAAGGA | GCTTGGAGCAAGTGCTGTGA |
| 18S (L33452) | ACCACATCCAAGGAAGGCAGCAGGC | CGGAGAGGAGCATGAGAAAC | CGTGCCAGGAGTGGGTAATTT |

Given are: Gene ID, EMBL or NCBI gene Identifier; Taqman probe, sense primer and antisense primer sequences. All sequences are given 5' to 3'. Legend: Hsp27 (AJ625244); Hsp 70 (AJ624049); Superoxide dismutase(FM177867.1); Catalase (AY580271.1); Glutathione-S-Transferase (AF527010.1); Caspase (HQ424451.1) P53(KC545827.1) Topoisomerase (AF227976.1) DNA Ligase (AJ624686.1) beta tubulin (AJ516796) RiboL27 (AJ625928); Actin (AJ625116); 18S (L33452).
